# Evolution and prognosis of tricuspid and mitral regurgitation following cardiac implantable electronic devices: a systematic review and meta-analysis

**DOI:** 10.1093/europace/euae143

**Published:** 2024-05-30

**Authors:** Matthew F Yuyun, Jacob Joseph, Sebhat A Erqou, Scott Kinlay, Justin B Echouffo-Tcheugui, Adelqui O Peralta, Peter S Hoffmeister, William E Boden, Hirad Yarmohammadi, David T Martin, Jagmeet P Singh

**Affiliations:** Department of Medicine, VA Boston Healthcare System, 1400 VFW Parkway, West Roxbury, MA 02132, USA; Department of Medicine, Harvard Medical School, 25 Shattuck St, Boston, MA 02115, USA; Department of Medicine, Boston University Chobanian and Avedisian School of Medicine, 72 E Concord St, Boston, MA 02118, USA; Department of Medicine, VA Boston Healthcare System, 1400 VFW Parkway, West Roxbury, MA 02132, USA; Department of Medicine, VA Providence Healthcare System, 830 Chalkstone Ave, Providence, RI 02908, USA; Department of Medicine, Brown University, 1 Prospect Street, Providence, RI 02912, USA; Department of Medicine, VA Providence Healthcare System, 830 Chalkstone Ave, Providence, RI 02908, USA; Department of Medicine, Brown University, 1 Prospect Street, Providence, RI 02912, USA; Department of Medicine, VA Boston Healthcare System, 1400 VFW Parkway, West Roxbury, MA 02132, USA; Department of Medicine, Harvard Medical School, 25 Shattuck St, Boston, MA 02115, USA; Department of Medicine, Boston University Chobanian and Avedisian School of Medicine, 72 E Concord St, Boston, MA 02118, USA; Department of Medicine, Brigham and Women’s Hospital, 75 Francis St, Boston, MA 02115, USA; Department of Medicine, Johns Hopkins University School of Medicine, 733 N Broadway, Baltimore, MD 21205, USA; Department of Medicine, VA Boston Healthcare System, 1400 VFW Parkway, West Roxbury, MA 02132, USA; Department of Medicine, Harvard Medical School, 25 Shattuck St, Boston, MA 02115, USA; Department of Medicine, Boston University Chobanian and Avedisian School of Medicine, 72 E Concord St, Boston, MA 02118, USA; Department of Medicine, VA Boston Healthcare System, 1400 VFW Parkway, West Roxbury, MA 02132, USA; Department of Medicine, Harvard Medical School, 25 Shattuck St, Boston, MA 02115, USA; Department of Medicine, Boston University Chobanian and Avedisian School of Medicine, 72 E Concord St, Boston, MA 02118, USA; Department of Medicine, VA Boston Healthcare System, 1400 VFW Parkway, West Roxbury, MA 02132, USA; Department of Medicine, Harvard Medical School, 25 Shattuck St, Boston, MA 02115, USA; Department of Medicine, Boston University Chobanian and Avedisian School of Medicine, 72 E Concord St, Boston, MA 02118, USA; Department of Medicine, Columbia University Irving Medical Center, 177 Fort Washington Avenue, New York, NY 10032, USA; Department of Medicine, Harvard Medical School, 25 Shattuck St, Boston, MA 02115, USA; Department of Medicine, Brigham and Women’s Hospital, 75 Francis St, Boston, MA 02115, USA; Department of Medicine, Harvard Medical School, 25 Shattuck St, Boston, MA 02115, USA; Department of Medicine, Massachusetts General Hospital, 55 Fruit St, Boston, MA 02114, USA

**Keywords:** Tricuspid regurgitation, Mitral regurgitation, Pacemaker, Implantable cardioverter defibrillator, Conduction system pacing, Cardiac resynchronization therapy, Leadless pacemaker

## Abstract

**Aims:**

Significant changes in tricuspid regurgitation (TR) and mitral regurgitation (MR) post-cardiac implantable electronic devices (CIEDs) are increasingly recognized. However, uncertainty remains as to whether the risk of CIED-associated TR and MR differs with right ventricular pacing (RVP) via CIED with trans-tricuspid RV leads, compared with cardiac resynchronization therapy (CRT), conduction system pacing (CSP), and leadless pacing (LP). The study aims to synthesize extant data on risk and prognosis of significant post-CIED TR and MR across pacing strategies.

**Methods and results:**

We searched PubMed, EMBASE, and Cochrane Library databases published until 31 October 2023. Significant post-CIED TR and MR were defined as ≥ moderate. Fifty-seven TR studies (*n* = 13 723 patients) and 90 MR studies (*n* = 14 387 patients) were included. For all CIED, the risk of post-CIED TR increased [pooled odds ratio (OR) = 2.46 and 95% CI = 1.88–3.22], while the risk of post-CIED MR reduced (OR = 0.74, 95% CI = 0.58–0.94) after 12 and 6 months of median follow-up, respectively. Right ventricular pacing via CIED with trans-tricuspid RV leads was associated with increased risk of post-CIED TR (OR = 4.54, 95% CI = 3.14–6.57) and post-CIED MR (OR = 2.24, 95% CI = 1.18–4.26). Binarily, CSP did not alter TR risk (OR = 0.37, 95% CI = 0.13–1.02), but significantly reduced MR (OR = 0.15, 95% CI = 0.03–0.62). Cardiac resynchronization therapy did not significantly change TR risk (OR = 1.09, 95% CI = 0.55–2.17), but significantly reduced MR with prevalence pre-CRT of 43%, decreasing post-CRT to 22% (OR = 0.49, 95% CI = 0.40–0.61). There was no significant association of LP with post-CIED TR (OR = 1.15, 95% CI = 0.83–1.59) or MR (OR = 1.31, 95% CI = 0.72–2.39). Cardiac implantable electronic device–associated TR was independently predictive of all-cause mortality [pooled hazard ratio (HR) = 1.64, 95% CI = 1.40–1.90] after median of 53 months. Mitral regurgitation persisting post-CRT independently predicted all-cause mortality (HR = 2.00, 95% CI = 1.57–2.55) after 38 months.

**Conclusion:**

Our findings suggest that, when possible, adoption of pacing strategies that avoid isolated trans-tricuspid RV leads may be beneficial in preventing incident or deteriorating atrioventricular valvular regurgitation and might reduce mortality.

## Graphical Abstract

**Graphical Abstract:**
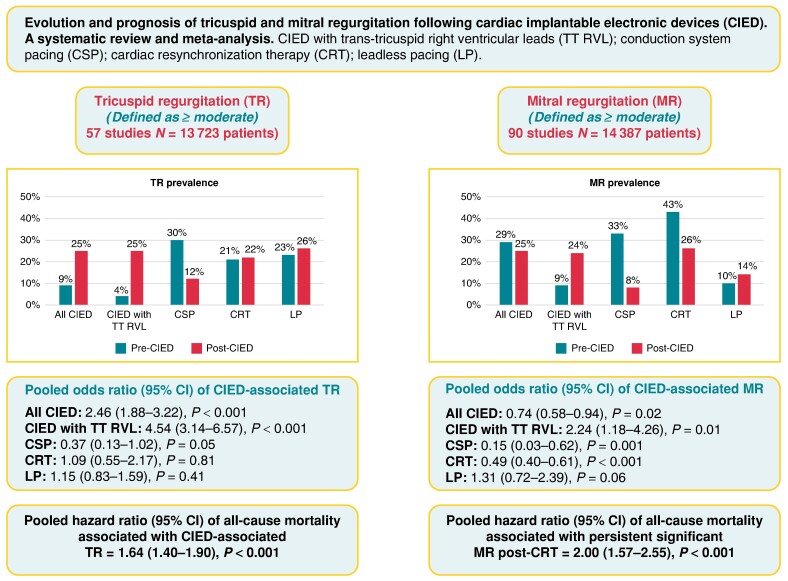

**What’s new?**

- Right ventricular pacing via cardiac implantable electronic device (CIED) with trans-tricuspid right ventricular (RV) leads was associated with increased risk of both significant post-CIED tricuspid regurgitation (TR) and mitral regurgitation (MR). The risk of TR more than quadrupled, while the risk of MR more than doubled following CIED with trans-tricuspid RV lead pacing.
- Conduction system pacing significantly reduced the risk of CIED-related MR.
- Cardiac resynchronization therapy considerably reduced the risk of secondary MR, but did not significantly impact the risk of TR. The reduction in secondary MR post- cardiac resynchronization therapy (CRT) was comparable across a wide range of quantitative parameters used for reporting mitral regurgitation severity.
- Leadless pacing did not significantly alter the risk of TR and MR post-CIED.
- Post-CIED TR was independently associated with poor survival, and persistence of significant MR post-CRT was independently associated with a two-fold increased risk of all-cause mortality.

## Introduction

Tricuspid regurgitation (TR) and mitral regurgitation (MR) whether structural or functional are independently associated with poor survival.^1,2^ Cardiac implantable electronic devices (CIEDs) are associated with new or worsening TR, and the prevalence of TR is higher in patients with CIED compared with controls.^3–8^ However, uncertainty remains as to whether the risk of significant TR is different following transvenous right ventricular pacing (RVP) via CIED with trans-tricuspid RV leads, compared with conduction system pacing (CSP), cardiac resynchronization therapy (CRT), and leadless pacing (LP). The first objective of this systematic review and meta-analysis was therefore to determine the risk of new significant TR defined as ≥ moderate TR between different pacing strategies or device types and the impact of this post-CIED TR on all-cause mortality.

While some studies have suggested that RVP via CIED with trans-tricuspid RV leads is associated with new or worsening MR,^9–13^ others have not,^14–18^ and the impact of other pacing strategies such as CSP and LP on MR remains unknown. While narrative reviews have reported that up to 40% of patients who have conventional indications for CRT have significant secondary MR at baseline^19^ and that CRT reduces systolic MR by ∼40%,^20,21^ the magnitude of benefit remains controversial, and a quantitative synthesis of the extant data on the effects of CRT on secondary MR is lacking. Hence, the second objective of this study was to determine the risk of new significant MR defined as ≥ moderate MR post-CIED implantation and the impact of significantly persistent MR post-CRT on all-cause mortality. Contemporary pacing guidelines increasingly favour CSP over traditional RVP via CIED with trans-tricuspid RV leads.^22,23^ Identifying the pacing strategies that are least likely to be associated with atrioventricular valvular insufficiency might potentially have beneficial clinical implications.

## Methods

This study was registered with the international prospective register of systematic reviews, PROSPERO (registration number CRD42021274269).

### Search strategy

PubMed/MEDLINE, EMBASE, and Cochrane Library databases were systematically searched to identify all relevant English language studies restricted to human adults published from inception until 31 October 2023 (see Supplementary material for search terms).

### Inclusion criteria

The inclusion criteria consisted of longitudinal studies reporting on patients with baseline pre-CIED [permanent pacemaker (PPM) or implantable cardioverter defibrillator (ICD), or CRT devices] assessment of TR or MR and follow-up post-CIED assessment of TR and MR by echocardiography. Included were studies that reported on TR or MR in a binary or categorical fashion as presence or absence of significant TR or MR defined as new ≥ moderate TR or MR and/or continuous quantitative measures of TR or MR post-CIED compared with the pre-CIED.

### Exclusion criteria

Excluded were non-English language studies lacking an English-translated version, studies that did not report clear assessment of TR or MR pre-CIED and post-CIED, and studies that included tricuspid or mitral valve repair or replacement, or trans-catheter percutaneous procedures such as MitraClip and other exclusion criteria stated in Supplementary material online, *Figure S1*.

### Outcomes

The main outcomes were as follows: (i) Prevalence of significant TR or MR post-CIED compared with prevalence at baseline pre-CIED. (ii) Risk of significant TR or MR post-CIED estimated using pooled odds ratio (OR) and 95% confidence interval (CI) for categorical data. (iii) For continuous or quantitative measures used to assess TR or MR, we used standardized mean difference (SMD) of change in post-CIED compared with pre-CIED TR and MR values. (iv) Comparison of the risk of TR post-CIED between ICD and PPM. (iv) Finally, the impact of post-CIED significant TR or MR on all-cause mortality during follow-up.

### Quality assessment of studies

The Newcastle–Ottawa Scale (NOS) was used for assessing the quality of non-randomized studies in meta-analyses. We categorized the studies according to NOS as follows: 0–3 = poor quality, 4–7 = fair quality, and 8–9 = good quality (see last column in Supplementary material online, *Tables S1* and *S2*).

### Data extraction

Two authors (M.F.Y. and S.A.E.) extracted the data independently using standardized forms containing pre-defined demographic and clinical information. Discrepancies were resolved by consensus.

### Statistical analysis

The pooled prevalence and OR and 95% CI of significant TR or MR for binary data and SMD (95% CI) for each continuous quantitative measure of TR or MR pre-CIED and post-CIED were calculated using random-effects model meta-analyses (DerSimonian–Laird), as some degree of heterogeneity was anticipated *a priori.* The pooled SMD were considered clinically significant for quantitative reduction of secondary TR or MR post-CIED compared with pre-CIED if the value was < 0 and the 95% CI did not cross 0. Hazard ratios and 95% CI for the risk of mortality associated with post-CIED TR or MR were also computed.

Between-study variation in ORs or HRs for categorical data and SMD for continuous data across studies that were attributable to heterogeneity was assessed by *I*^2^ statistic. Publication bias was assessed by eye-balling funnel plots and contour-enhanced funnel plots and Egger’s test of bias. Where there was suggestion of publication bias, this was further explored using trim and fill analysis. Analyses were performed with STATA software 18 (*Stata Corp, Texas*).

## Results

Of the 12 435 initial citations identified through literature search, 130 studies with data on pre- and post-CIED implantation echocardiographic measurements of TR and MR were included. Of these, **57 studies** with 68 data points (***n* = 13 723 patients**)^5,6,9,10,12,14–18,24–70^ assessed TR, while **90 studies** with 104 data points (***n* = 14 387 patients**)^9–12,14–18,30,32,37,38,41,60,65,68–141^ assessed MR. Retained studies for TR and MR were not mutually exclusive as 17 of these reported both TR and MR changes from pre- to post-CIED (*see consort diagram on*  Supplementary material online, *Figure S1*).

### Study characteristics

The characteristics of participants for studies that evaluated TR are shown in Supplementary material online, *Table S1*, and MR in Supplementary material online, *Table S2*. For TR, the pooled mean age weighted by study population was 67.6 years, and 53% were men. Pooled common comorbidities were hypertension (60.3%), coronary artery disease (40.4%), hyperlipidaemia 43.9%, atrial fibrillation (39.3%), diabetes mellitus (30.4%), chronic kidney disease (20.8%), cerebrovascular accident (13.9%), and chronic airway disease (13.8%), and the baseline left ventricular ejection fraction (LVEF) was 49.9%. For TR, the PPM device accounted for 60.5% of all the CIED implants, ICD devices 19.5%, and CRTP/CRTD devices 20.0%. For MR, the pooled mean age was 64.6 (6.2) years, 74.3% were men, and 21.1% of all devices were PPM or ICD, while the rest were CRTP/CRTD, pooled LVEF 24.5%, and ischaemic heart disease 51.6%, but significant missing data prevented pooling other comorbidities.

### Duration of follow-up for the assessment of tricuspid regurgitation and mitral regurgitation post-cardiac implantable electronic device

The pooled median duration of follow-up post-CIEDs for TR studies at which point TR was assessed was 12 months [interquartile range (IQR) 8.9–20 months], while for MR it was 6 months (IQR 3–12 months). The median duration of follow-up during at which all-cause mortality associated with persistently significant TR post-CIED was ascertained across TR studies was 53 months (IQR 39–70 months), while for MR it was 38 months (IQR 32–48 months).

### Risk of tricuspid regurgitation and mitral regurgitation post-cardiac implantable electronic device

#### All cardiac implantable electronic device implants and risk of tricuspid and mitral regurgitation

**Figure 1:**
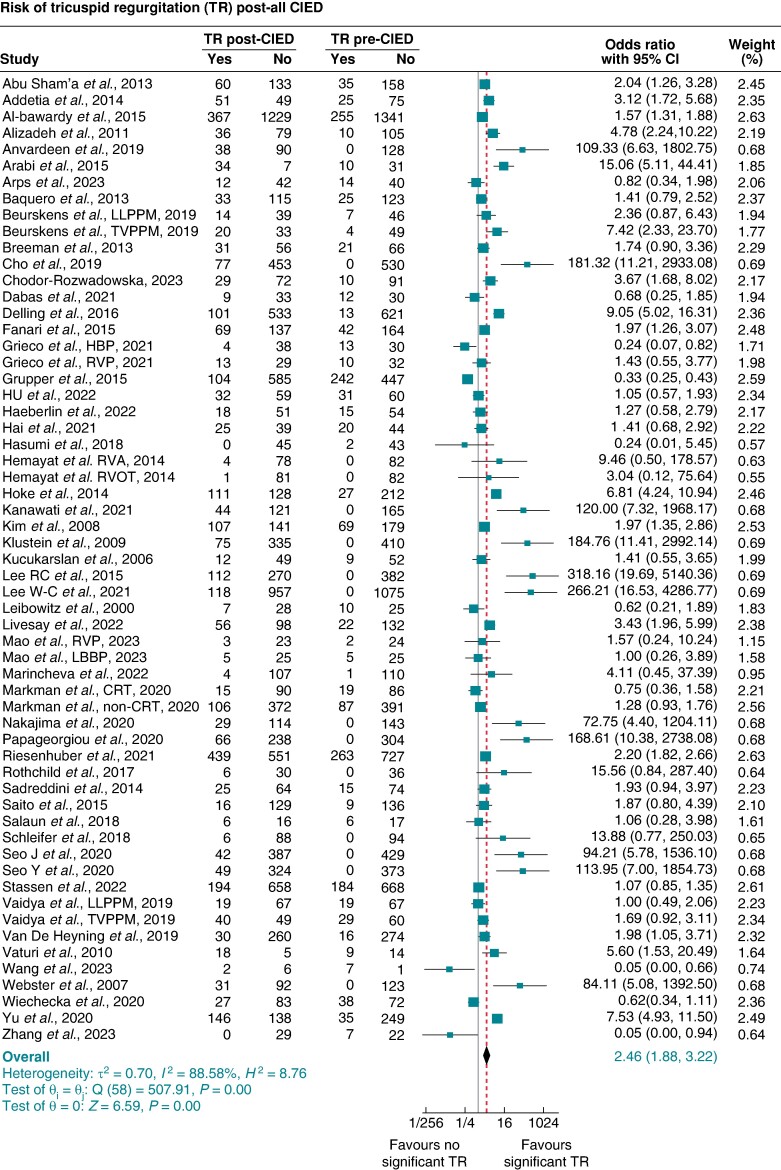
Pooled odds ratio (OR) and 95% confidence interval (95% CI) of significant tricuspid regurgitation (≥ moderate TR) post-CIED compared with pre-CIED implantation for all devices combined. CIED, cardiac implantable electronic device.

For TR, 53 studies^5,6,9,10,12,14–18,24–29,31–40,42–59,61–67,69,70^ produced 59 data points to pool the proportion and risk of significant TR post-CIED compared with pre-CIED implantation (see Supplementary material online, *Table S3*). The pooled prevalence of significant TR at baseline pre-CIED was 9% (95% CI 8–10%) (see Supplementary material online, *Figure S2*), increasing by more than two-fold to 25% (95% CI 21–28%) post-CIED, corresponding to an absolute incidence of 16% (see Supplementary material online, *Figure S3*). The pooled OR and 95% CI of new ≥ moderate TR post-CIED were 2.46 (1.90–3.20), *P* < 0.001, *I*^2^ 88.71%, and Egger’s test *P*-value < 0.001 (*Figure 1*).

**Figure 2:**
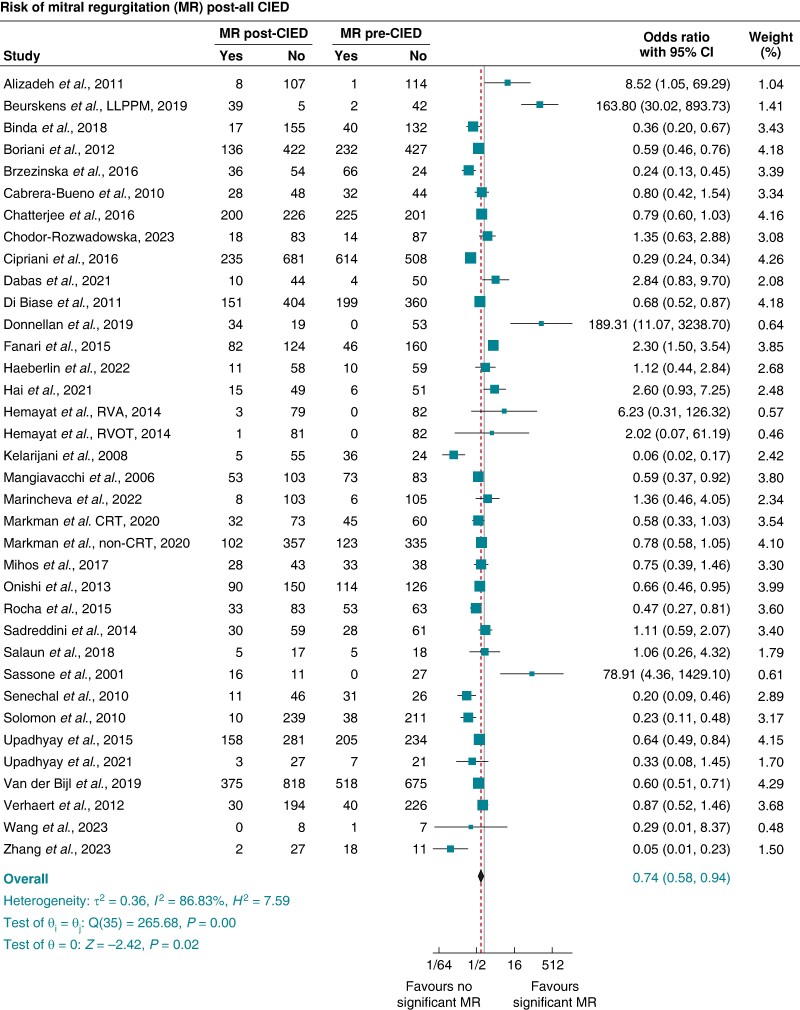
Pooled odds ratio (OR) and 95% confidence interval (95% CI) of significant mitral regurgitation (≥ moderate MR) post-CIED compared with pre-CIED implantation for all devices combined. CIED, cardiac implantable electronic device.

For MR, 34 studies^9–12,14–18,32,33,37,38,65,70,78,80,83–86,89,97,106,111,114,120,121,124,131,133,134,140,141^ produced 36 data points to pool the proportion and risk of significant MR post-CIED compared with pre-CIED implantation (see Supplementary material online, *Table S4*). The pooled prevalence of significant MR at baseline pre-CIED was 29% (95% CI 22–35%), decreasing to 25% (95% CI 20–30%) post-CIED. The pooled OR (95% CI) of significant MR associated with all CIED was 0.74 (0.58–0.94), *P* = 0.02, *I*^2^ 86.83%, and Egger’s test *P*-value < 0.0001 (*Figure 2*)

#### Right ventricular pacing via cardiac implantable electronic device with trans-tricuspid right ventricular leads and risk of tricuspid and mitral regurgitation

**Figure 3:**
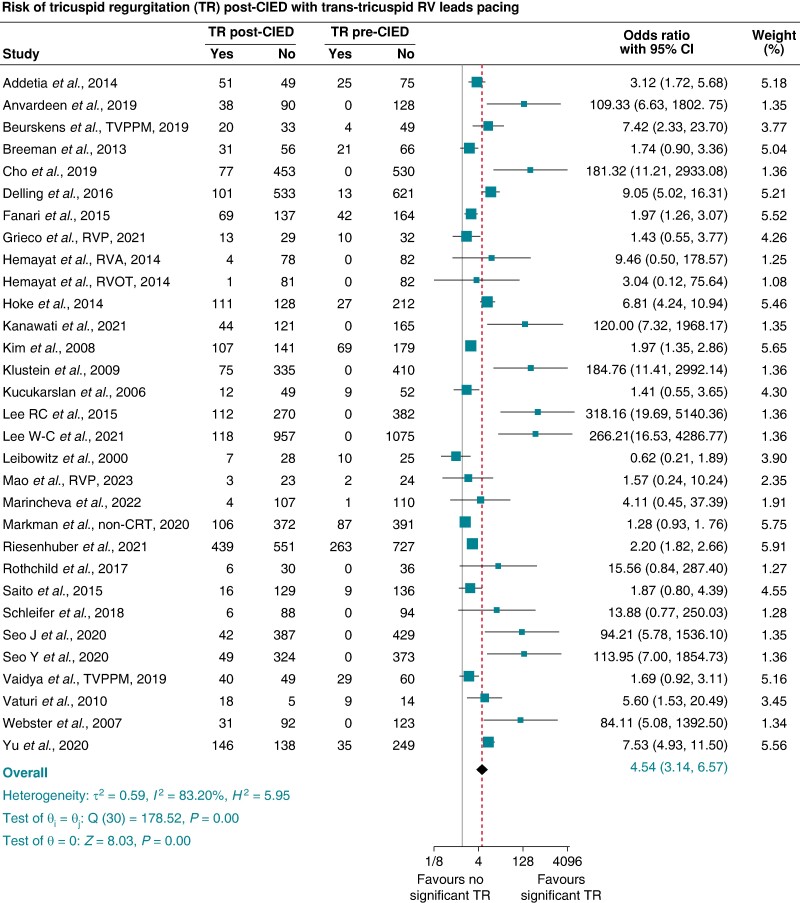
Risk of significant tricuspid regurgitation (≥ moderate TR) post-transvenous right ventricular pacing via CIED trans-tricuspid right ventricular leads compared with pre-CIED implantation [pooled odds ratio and 95% confidence interval (95% CI)]. CIED, cardiac implantable electronic device.

**Figure 4:**
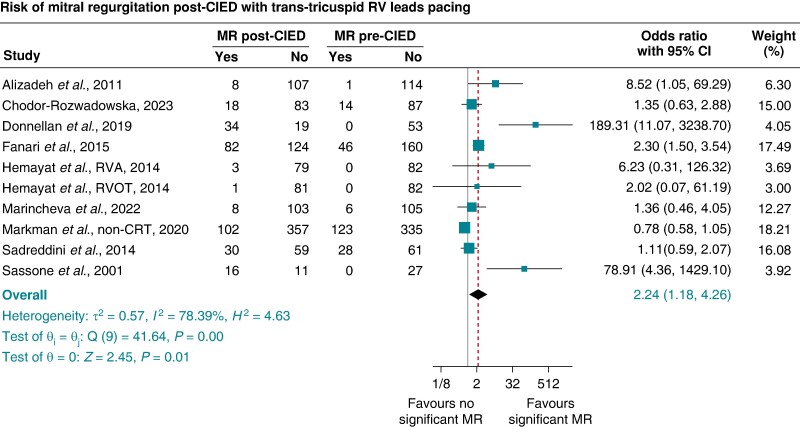
Risk of significant mitral regurgitation (≥ moderate MR) post-transvenous right ventricular pacing via CIED with trans-tricuspid RV leads compared with pre-CIED implantation (pooled odds ratio and 95% confidence interval). CIED, cardiac implantable electronic device.

Excluding LP, CSP, and any studies that included patients with CRT and leaving only RVP via CIED with trans-tricuspid RV lead studies, the pooled prevalence of significant TR pre-RVP was 4% (95% CI 3–5%), which increased significantly to 25% (95% CI 20–30%) post-RVP. The pooled OR (95% CI) of significantly new TR associated with RVP via CIED with trans-tricuspid RV leads was 4.54 (3.14–6.57), *P* < 0.001, *I*^2^ 83.20%, and Egger’s test *P*-value < 0.001 (*Figure 3*), with results remaining significant after trim and fill analysis. The pooled prevalence of significant MR pre-RVP was 9% (95% CI 5–14%), which increased to 24% (95% CI 14–33%) post-RVP. The pooled OR (95% CI) of significant MR associated with RVP via CIED with trans-tricuspid RV leads was 2.24 (1.18–4.26), *P* = 0.01, *I*^2^ 78.39%, and Egger’s test *P*-value = 0.0005 (*Figure 4*).

#### Conduction system pacing and risk of tricuspid and mitral regurgitation

**Figure 5:**
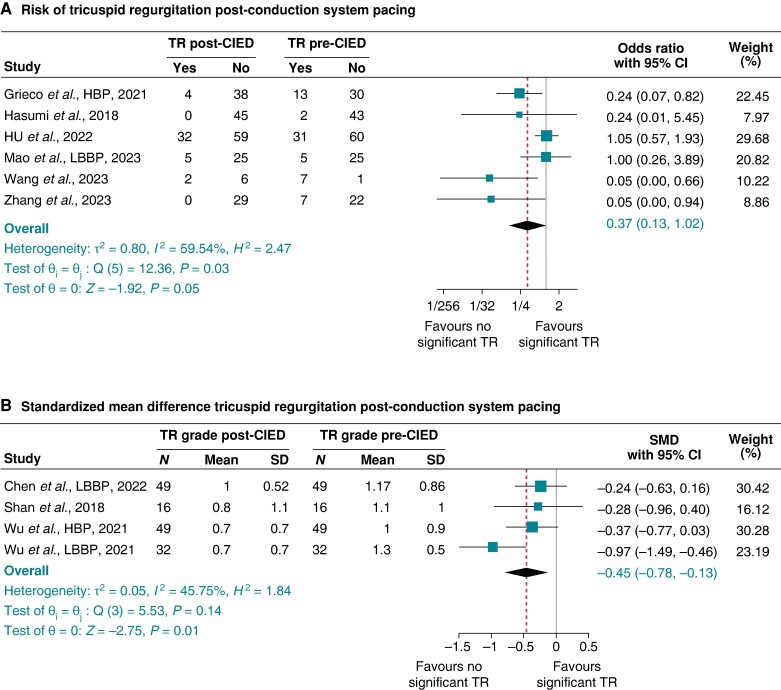
Tricuspid regurgitation post-conduction system pacing. (*A*) Odds ratio for studies that provided binary data and (*B*) standardized mean difference (SMD) for studies that provided continuous data. HBP, His bundle pacing; LBBP, left bundle branch pacing.

**Figure 6:**
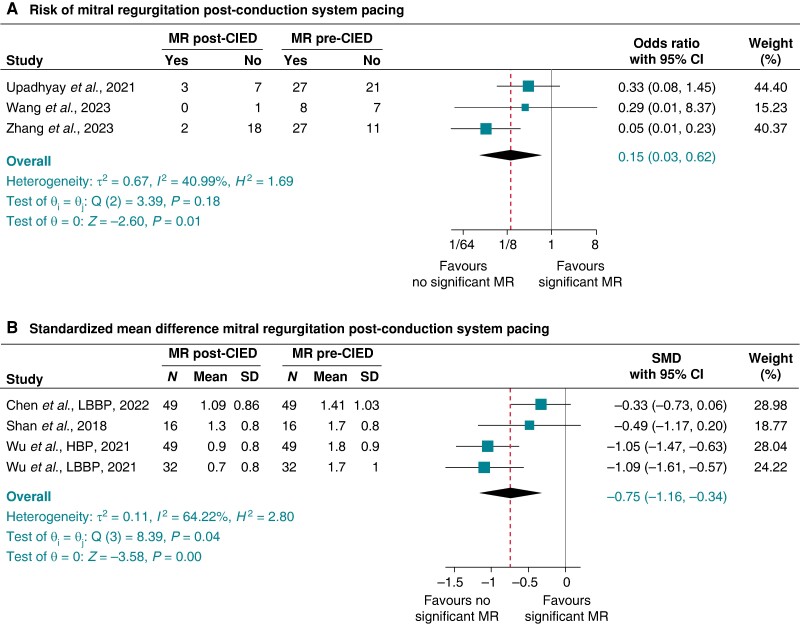
Mitral regurgitation post-conduction system pacing. (*A*) Odds ratio for studies that provided binary data and (*B*) standardized mean difference (SMD) for studies that provided continuous data. HBP, His bundle pacing; LBBP, left bundle branch pacing.

Excluding all studies with CIED with trans-tricuspid RV leads, LP, and any CRT with biventricular leads, 10 studies^30,34,36,39,41,50,60,65,68,70^ for TR and 7 studies^30,41,60,65,68,70,141^ for MR exclusively of patients implanted with CSP (His bundle or left bundle branch or left ventricular septal pacing) were retained for this sub-group analysis. For TR studies with data in the binary format, the pooled prevalence of significant TR pre-CSP was 30% (95% CI 13–47%), which reduced post-CSP to 12% (95% CI 3–21%): pooled OR 0.37 (95% CI 0.13–1.02), *P* = 0.05, *I*^2^ 59.54%, and Egger’s test *P*-value = 0. 0.0042 (*Figure 5A*). There was significant reduction in MR post-CSP at 8% (1–14%) compared with pre-CSP at 33% (4–62%): pooled OR 0.15 (95% CI 0.03–0.62), *P* = 0.001, *I*^2^ 40.99%, and Egger’s test *P*-value = 0.7898 (*Figure 6A*). Findings from studies with continuous data format for TR are shown in *Figure 5B* and MR in *Figure 6B*. A negative SMD and 95% CI in the negative range indicated significant reduction in TR or MR grade post-CIED compared with pre-CRT.

#### Cardiac resynchronization therapy and risk of tricuspid and mitral regurgitation

**Figure 7:**
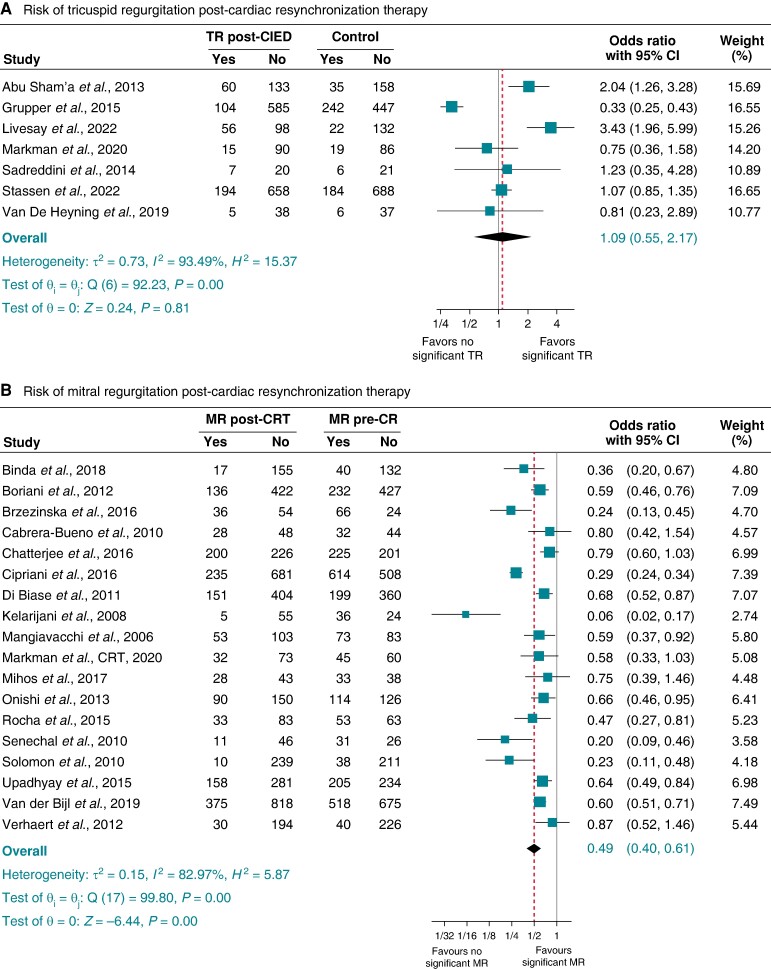
Risk of significant tricuspid regurgitation (TR) (*A*) and secondary mitral regurgitation (MR) (*B*) post-cardiac resynchronization compared with pre-cardiac resynchronization therapy (CRT).

*Prevalence and risk of significant TR post-CRT:* Seven studies^17,18,35,49,59,61,63^ consisting exclusively of CRT patients with biventricular leads examined the TR incidence post-CRT implantation compared with pre-implant. The pooled proportion of significant TR pre-CRT was 21% (95% CI 14–27%) and post-CRT 22% (95% CI 16–28%): pooled OR (95% CI) of significant TR post-CRT compared with pre-CRT of 1.09 (0.55–2.17), *P* = 0.81, *I*^2^ 93.49%, and Egger’s test *P*-value = 0.8323 (*Figure 7A*).

*Prevalence and risk of significant MR post-CRT compared with pre-CRT: binary data*—Amongst the 18 studies^17,78,80,83–86,89,97,106,111,114,120,121,124,131,133,134^ with binary data exclusively of patients implanted with biventricular pacing device in the setting of heart failure with reduced ejection fraction (HFrEF), the pooled prevalence of significant MR pre-CRT was 43% (95% CI 36–49%), which significantly reduced post-CRT to 26% (95% CI 21–32%) (see Supplementary material online, *Figures S4* and *S5*): pooled OR (95% CI) of 0.49 (0.40–0.61), *P* < 0.001, *I*^2^ 82.97%, and Egger’s test *P*-value = 0.0082 (*Figure 7B*). Of note, some studies^41,75,77–80,84,86,89,95,97,104,111,112,114,116,118,120–125,131–133^ reported the proportion of patients with improvement in MR and the summarized proportion of patients with at least one grade improvement in secondary MR post-CRT compared with pre-CRT measures weighted by study population was 45.5% (standard deviation 12.4%).

*Mitral regurgitation quantitative parameters post-CRT compared with pre-CRT: continuous data*—The binary data findings were also robustly replicated in the remaining studies^30,68,71–76,79,81,82,87,88,90–96,98–102,104,105,107–111,113,116–119,121–130,132,134–139,142^ that reported MR severity quantified using continuous parameters: MR grade, effective regurgitant orifice area (EROA), regurgitant volume, regurgitant fraction, mitral regurgitant jet area, ratio of mitral regurgitant jet area to left atrial area, and vena contracta (**see**  Supplementary material online, *Tables S5*–*S10* and *Figures S6*–*S9*).

#### Leadless pacing and risk of tricuspid and mitral regurgitation

**Figure 8:**
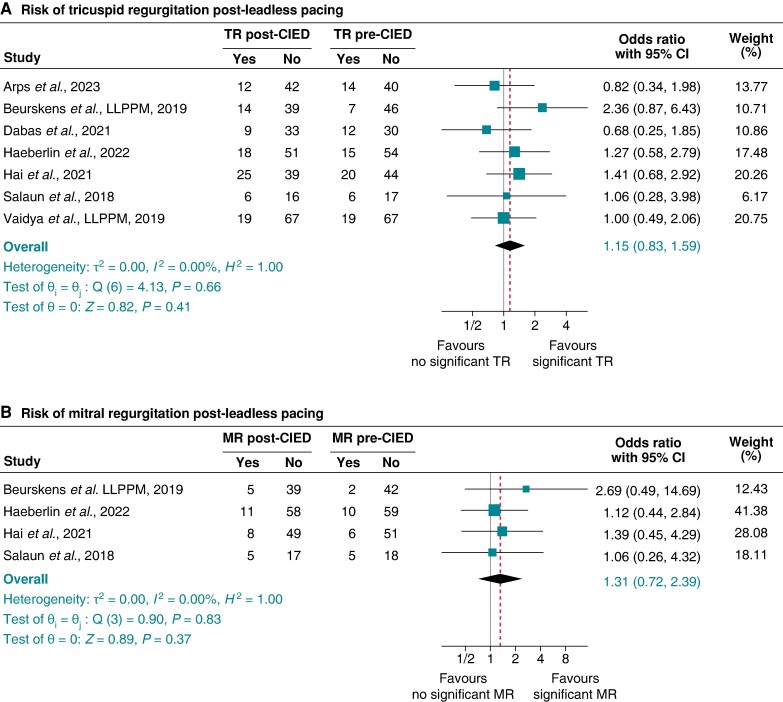
Risk of tricuspid regurgitation (TR) (*A*) and mitral regurgitation (MR) (*B*) after leadless permanent pacemaker implantation.

For TR, after excluding all transvenous RVP studies, CSP, and CRT, seven studies^12,14,27,33,37,38,62^ exclusively of patients implanted with leadless pacemakers were retained for this sub-group analysis, with pooled OR 1.15 (95% 0.83–1.59), *P* = 0.410, *I*^2^ 0.00%, and Egger’s test *P*-value = 0.9166 (*Figure 8A*). For MR, in four studies,^12,14,37,38^ the pooled OR was 1.31 (95% CI 0.72–2.39), *P* = 0.37, *I*^2^ 0.00%, and Egger’s test *P*-value = 0.9468 (*Figure 8B*).

#### Implantable cardioverter defibrillator vs. permanent pacemaker and risk of post-cardiac implantable electronic device tricuspid regurgitation

The OR (95% CI) of post-CIED TR in ICD devices compared with pacemaker devices was not significant at 1.14 (0.74–1.75), *P* = 0.55, *I*^2^ 63.07%, and Egger’s test *P*-value = 0.9772 (see Supplementary material online, *Figure S10*).

#### Tricuspid regurgitation post-cardiac implantable electronic device and all-cause mortality

**Figure 9:**
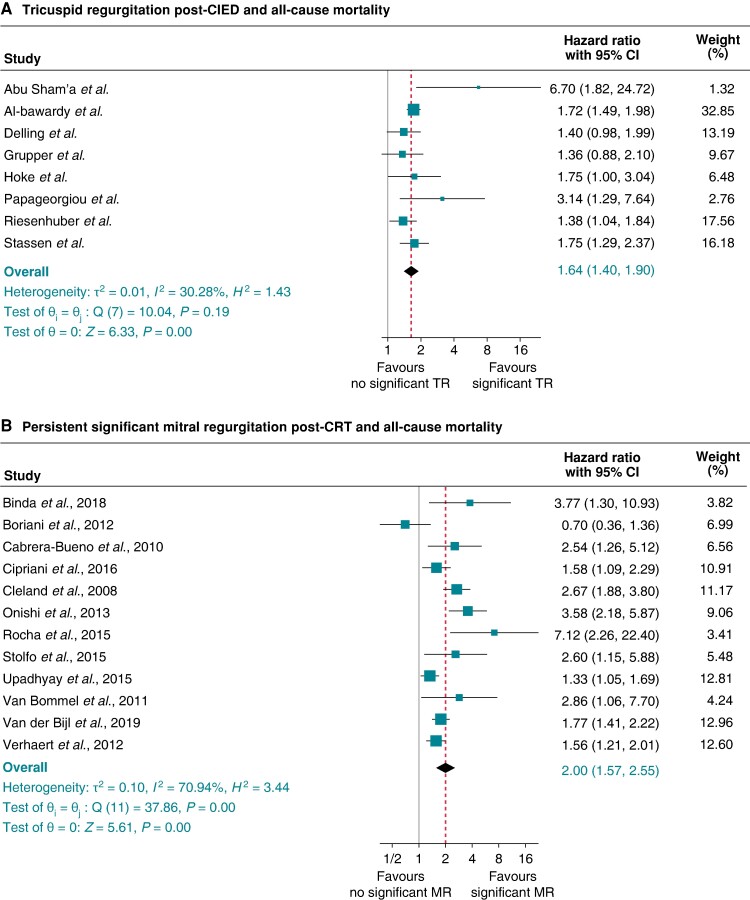
Pooled hazard ratio (95% confidence interval) of all-cause mortality associated with presence vs. absence of persistent significant tricuspid regurgitation post-all cardiac implantable electronic devices (CIEDs) (*A*) and secondary mitral regurgitation post-cardiac resynchronization therapy (CRT) (*B*).

Eight studies^5,6,35,40,52,53,59,61^ were included in the meta-analysis of CIED-associated TR and all-cause mortality (see Supplementary material online, *Table S11*). Pooled adjusted HR (95% CI) of all-cause mortality after a median follow-up of 53 months (IQR 39–70 months) associated with significant TR post-CIED was 1.64 (1.40–1.90), *P* < 0.001, *I*^2^ 30.28%, and Egger’s test *P*-value = 0.0884 (*Figure 9A*).

#### Persistent secondary mitral regurgitation post-cardiac resynchronization therapy and all-cause mortality

Twelve studies^78,80,84,86,87,114,120,129,131–134^ evaluated this risk. The pooled adjusted hazard ratio of all-cause mortality after a median follow-up 38 months, associated with significant secondary MR that persisted during the assessment at median follow-up post-CRT of 6 months compared with no significant secondary MR, was 2.00 (95% CI 1.57–2.55; *P* < 0.001), *I*^2^ 70.94%, and Egger test *P*-value 0.0991 (*Figure 9B*).

#### Publication bias and small-study effects

Funnel plots of OR against their standard error for TR are shown in Supplementary material online, *Figure S11*, and MR in Supplementary material online, *Figure S12*. Within each of these figures, panels A to E depict various pacing strategies, while panel F shows the funnel plot derived from the HR of all-cause mortality. Contour-enhanced funnel plots are shown in Supplementary material online, *Figure S13* (panels A to C for TR and D to F for MR). For any asymmetry in funnel plot or discordant distribution of studies in contour-enhanced funnel plot or significant Egger’s test *P*-value observed in the various analyses, trim and fill analysis showed no significant change in results between the observed and the observed + imputed, suggesting less likelihood of publication bias, though the possibility of small-study effects remained when Egger’s test *P*-value was significant.

## Discussion

Amongst the 13 723 patients (TR) and 14 387 patients (MR) assessed for the risk of CIED-associated TR and MR, this systematic review and meta-analysis observed the following: (i) All CIED grouped together were associated with a more than two-fold increased risk of significant TR, while the risk of MR post-all CIED was reduced likely due the high number of CRT studies in whom post-CIED MR significantly decreased. (ii) RVP via CIED with trans-tricuspid RV leads was associated with increased risk of both significant post-CIED TR and MR. The risk of TR more than quadrupled, while the risk of MR more than doubled following implantation of CIED with trans-tricuspid RV leads. (iii) Conduction system pacing significantly reduces the risk of post-CIED MR. (iv) Cardiac resynchronization therapy considerably reduced the risk of secondary MR, but did not significantly impact the risk of TR. The CRT-associated reduction in MR was comparable across a wide range of quantitative parameters used for reporting MR severity such as MR grade, EROA, regurgitant volume, regurgitant fraction, mitral regurgitant jet area, ratio of mitral regurgitant jet area to left atrial area, and vena contracta. (iv) Leadless pacing had a risk-neutral effect on both incident TR and MR post-CIED. (vi) There was no significant difference in post-CIED TR occurrence between ICD and PPM devices. (vii) Post-CIED TR was independently associated with poor survival, and persistence of significant MR post-CRT was independently associated with a two-fold increased risk of all-cause mortality. To the best of our knowledge, this study is the first comprehensive meta-analysis to assess the risk of atrioventricular valvular regurgitation post-CIED across all pacing strategies and device types.

This meta-analysis revealed that significant TR (≥ moderate) increased considerably from 9% pre-CIED to 25% post-CIED. Of note, given that some studies variably excluded patients with any or significant TR at baseline, the observed pre-CIED prevalence of TR in this study is likely an underestimate of real-world prevalence. Prior meta-analyses concluded that the overall incidence of at least one grade increase in TR post-CIED was approximately 25%, and approximately 10% for at least two grades increase.^3,4,8^ Our findings are similar to those of a prior study that observed OR of new or worsening CIED-associated TR of 2.44 (95% CI 1.58–3.77) when compared with patients without CIED.^4^ Nonetheless, despite adopting a different methodology, the findings of this meta-analysis complement those of these prior studies with a much larger sample size and additional sub-group analyses across different pacing strategies. We found no significant difference in the rate of occurrence of CIED-associated TR between ICD and PPM device recipients, which was consistent with the findings of others,^9,25,28,40,42,46,56,67^ despite two individual studies reporting higher incidence of TR post-ICD devices compared with pacemakers.^43,52^ This suggests that the size of the lead might not matter. Pacing factors such as the percentage of RV pacing have not been shown to affect the incidence of post-CIED TR.^25,46,55^ Right ventricular pacing site in relation to CIED-associated TR has yielded inconsistent results.^56,69^

This meta-analysis examined non-RV pacing strategies and risk of TR and MR and showed significant benefits of CSP in reducing the risk of MR in both binary and continuous data analyses, as well as TR in continuous data analysis with significant reduction in TR grade. Contemporary guidelines recommend offering patients CSP, when possible, given that large observational studies have shown its significant superiority over RVP as well as over biventricular pacing with respect to echocardiographic outcomes, all-cause mortality, and heart failure hospitalization.^22,23^ The results of this present meta-analysis suggest that in addition to the above, CSP should also be considered over RVP via CIED with trans-tricuspid RV leads to reduce the risk of MR and potentially reduce the risk of TR. The benefit of His bundle pacing (HBP) on post-CIED TR has been previously highlighted with improvement seen in TR grade after HPB for CRT as well as for the atrioventricular block.^143^ Conduction system pacing involves a trans-tricuspid lead, but it remains uncertain why the risk of TR might be less than with conventional RVP. Whether this possible risk reduction in TR might stem from less lead mechanical pressure exerted on the tricuspid valve apparatus by the lighter small diameter (1.4 mm) Medtronic SureScan 3830 lead that has been widely used so far for CSP, compared with the ticker stylet-driven leads or other mechanisms, remains speculative. This is especially so as the size of the lead has not been shown to alter the risk of TR.^9,25,28,40,42,46,56,67^

Patients implanted with biventricular pacing devices did not witness any significant change in TR, and this is probably due to less ventricular dyssynchronous changes seen in CRT. These findings are supported by a few studies that showed that CRT compared with non-CRT devices is not associated with significant TR incidence.^18,49^ The absence of a significant risk of TR in patients with LP and the strong association with transvenous trans-valvular RVP suggest that the presence of trans-valvular leads might contribute to the pathophysiologic mechanism of TR post-RVP, and it is possible that a trans-tricuspid lead position rather than RVP *per se* leads to TR. It is also conceivable that some of the lack of association of LP with TR could be also driven by the mid-to-high septum placement of the leadless pacemakers. However, prior studies have shown conflicting findings between the RV pacing site and risk of TR.^56,69^

There is still palpable ambiguity surrounding mechanisms of pacing-induced TR. Various pathophysiologic phenomena have been suggested to interfere with tricuspid valve apparatus leading to TR. These include direct lead-related factors that can engender direct mechanical leaflet damage such as lead position (commissural vs. non-commissural), lead interference with leaflet mobility (lead impingement or adherence/fibrosis or lead entrapment or excessive lead slack), leaflet perforation, number of leads, lead thrombosis, and lead impact on sub-valvular apparatus, all potentially leading to impaired leaflet co-aptation and excursion leading to significant TR post-CIED.^7,8,24,25,69,144–146^ Non-lead-related factors such as atrioventricular dyssynchrony, right ventricular dyssynchrony, pacing-induced left ventricular dysfunction, increased pulmonary artery systolic pressure, RV dilatation, RV dysfunction, tricuspid valve annular dilatation, leaflet tethering, and mal-co-aptation, which eventually culminate to TR, have also been suggested.^7,31,64^ The risk factors of CIED-associated TR which are not unanimously found include pulmonary artery systolic pressure, lead duration, atrial fibrillation, pre-existing mild TR, diabetes, heart failure, and peripheral vascular disease.^31,63,144,147^

Over the past decade, small to medium size studies have accrued showing conflicting findings about RVP and MR.^9–12,14–18,32,37,38,140^ The findings from this meta-analysis, which pooled these studies together, appear to cement RVP via CIED with trans-tricuspid RV leads as an inducer of significant MR post-CIED. The possible mechanisms of pacing-induced MR were first suggested in experimental animal models, which observed that the development of MR was associated with LV dyssynchrony secondary to RVP, leading progressively to worsening LV contractility and increasing LV geometry and left ventricular dilation, mitral annular dilatation, and MR.^148^ Right ventricular pacing is known to be associated with pacing-induced cardiomyopathy,^149^ and functional MR is a now a well-known consequence of LV systolic dysfunction.^150^ Other mechanisms include AV dyssynchrony and adverse changes in the papillary muscle function.^151^ Given that CSP reduces electrical dyssynchrony, mechanical dyssynchrony, and the chances of left ventricular size and geometry derangements and improves systolic dysfunction,^152^ it is therefore understandable why it reduces the risk of functional MR as observed in this meta-analysis. However, like TR, the reason why LP does not lead to significant MR despite its RV location as observed in this study remains unclear. Unlike TR, the increasing frequency of RV pacing worsens MR.^9,140^

Improvement in functional MR post-CRT is likely due to an interplay of multiple complex and varied mechanisms leading to LV reverse remodelling ranging from reduction in left ventricular volumes with a decrease in mitral leaflet tenting angles and augmentation of trans-mitral pressure gradient attributable to increased and coordinated left ventricular contractility to improved coordination of the papillary muscle closing forces, which may facilitate effective mitral valve closure.^20,82,114,123,133,137,138^ Cardiac resynchronization therapy reduces regurgitant volume and papillary muscle tethering on mitral leaflets leading to improvement in leaflet co-aptation and resynchronization of atrioventricular and inter- and intra-ventricular contraction.^82,123,133^ Significant healthcare utilization costs have associated with CRT-non-response, highlighting the important role CRT plays in heart failure,^153^ and strategies on optimized implementation of CRT use have been proposed.^154^

## Limitations

This systematic review and meta-analysis had important limitations. First, the majority of included studies was observational cohort studies, many of which were retrospective with all the inherent biases and confounders associated with such a methodology as well as small-study effects. For any asymmetry in funnel plot or discordant distribution of studies in contour-enhanced funnel plot or significant Egger’s test *P*-value observed in the various analyses, trim and fill analysis showed no significant change in results between the observed and the observed + imputed, suggesting less likelihood of publication bias, which increases confidence in the findings. Second, the pooled baseline patient characteristics were widely different amongst the studies, due to inclusion of patients with structurally normal hearts at baseline (bradycardia indications) and patients with heart failure (in CRT studies) and might not be fully representative of each sub-group. Third, due to the high number of missing or non-uniformly reported co-variates across studies, it was not possible to reliably carry out a meta-regression to explore sources of heterogeneity in CIED-associated TR and MR or to assess possible correlates of CIED-associated TR and MR. Fourth, as MR is dynamic and may fluctuate prospectively due to various positive or negative remodelling drivers, it hard to ascribe all the credit of post-CRT secondary MR improvement to CRT, especially as we did not compare with the group without CRT.

## Conclusions

This meta-analysis revealed the deleterious effect of transvenous RVP via CIED with trans-tricuspid RV leads on the risk of significant CIED-associated TR and MR with no significant difference between ICD and PPM for TR risk and the beneficial effect of CSP for MR and maybe TR, as well as CRT for MR, while LP was associated with unchanged risk. It also revealed the association of CIED-associated or persistent post-CIED atrioventricular valvular regurgitation with lower survival. Based on these findings, opting for pacing strategies that avoid isolated trans-tricuspid RV leads, when possible, might offer protection against incident or deteriorating TR and MR and might even reduce pre-existing significant atrioventricular valvular insufficiency and potentially reduce the risk of all-cause mortality.

## Supplementary Material

euae143_Supplementary_Data

## Data Availability

All relevant data are within the manuscript and its online supplementary material. This is a systematic review and meta-analysis of published data. No data are held or will be held in a public repository. All the extracted data for this study can found in various tables within the online supplementary material.

## Supplementary material

Supplementary  material is available at *Europace* online.
