# Supplementary material for: Evolution and prognosis of tricuspid and mitral regurgitation following cardiac implantable electronic devices: a systematic review and meta-analysis
Source: Europace. 2024 May 30;26(7):euae143. doi: 10.1093/europace/euae143 (PMC11259857; doi:10.1093/europace/euae143)
Supplement: euae143_Supplementary_Data [file euae143_supplementary_data.docx]

**Evolution and Prognosis of Tricuspid and Mitral Regurgitation Following Cardiac Implantable Electronic Devices. A Systematic Review and Meta-analysis.**

**SUPPLEMENTARY MATERIAL**

**Methods:** Search strategy

Supplementary Figure 1: Consort Diagram Tricuspid and Mitral Regurgitation post-cardiac implantable electronic devices.

Supplementary Table 1: Baseline characteristics for tricuspid regurgitation post-cardiac implantable electronic devices (CIED).

Supplementary Table 2: Baseline characteristics for mitral regurgitation post-cardiac implantable electronic devices.

Supplementary Table 3: Tricuspid regurgitation post- cardiac implantable electronic devices and duration of follow-up.

Supplementary Table 4: Mitral regurgitation (MR) pre- and post- cardiac implantable electronic devices and duration of follow-up.

Supplementary Table 5: Mean grade of secondary mitral regurgitation (MR) pre- and post-cardiac resynchronization therapy.

Supplementary Table 6: Mean effective regurgitant orifice area (EROA) in mm^2^ of secondary mitral regurgitation (MR) pre- and post-cardiac resynchronization therapy.

Supplementary Table 7: Mean regurgitant volume (RV) and regurgitant fraction (RF) of secondary mitral regurgitation (MR) pre- and post-cardiac resynchronization therapy.

Supplementary Table 8: Mean mitral regurgitant jet area (MRJA) in cm^2^ of secondary MR pre- and post-cardiac resynchronization therapy.

Supplementary Table 9: Mean proportion of mitral regurgitant jet area to left atrial area (MRJA/LAA %) of secondary mitral regurgitation (MR) pre- and post-cardiac resynchronization therapy.

Supplementary Table 10: Mean vena contracta (VC) of secondary mitral regurgitation (MR) pre- and post-cardiac resynchronization therapy.

Supplementary Table 11: All-Mortality risk associated with significant TR post- cardiac implantable electronic devices.

Supplementary Table 12: All-Mortality risk associated with significant mitral regurgitation post-cardiac resynchronization therapy.

Supplementary Figure 2: Proportion (prevalence) of significant TR pre-CIED implantation

Supplementary Figure 3: Proportion (prevalence) of significant TR post-CIED implantation

Supplementary Figure 4: Proportion (prevalence) of significant MR pre-CRT implantation

Supplementary Figure 5: Proportion (prevalence) of significant MR post-CRT implantation

Supplementary Figure 6: Standardized mean difference (SMD) and 95% confidence interval (95% CI) in secondary mitral regurgitation (MR) grade *(6A)* and effective regurgitant orifice area (EROA*) (6B)* pre- and post-cardiac resynchronization therapy.

Supplementary Figure 7: Standardized mean difference (SMD) in mitral regurgitation (MR) regurgitant volume *(7A)*, and regurgitant fraction *(7B)* fraction pre- and post-cardiac resynchronization therapy.

Supplementary Figure 8: Standardized mean difference (SMD) and 95% confidence interval (95% CI) in secondary mitral regurgitation (MR) mitral regurgitant jet area (MRJA) *(8A)* & proportion of mitral regurgitant jet area to left atrial area (MRJA/LAA %) *(8B)* pre- and post-cardiac resynchronization therapy.

Supplementary Figure 9: Standardized mean difference (SMD) in mitral regurgitation (MR) vena contracta pre- and post-cardiac resynchronization therapy.

Supplementary Figure 10: Risk of significant Tricuspid regurgitation (TR) post-implantable cardioverter defibrillator (ICD) devices compared to permanent pacemakers (PPM).

Supplementary Figure 11: Funnel plots of studies included in meta-analysis of tricuspid regurgitation (TR) post-cardiac implantable electronic devices (CIED).

Supplementary Figure 12: Funnel plots of studies included in meta-analysis of mitral regurgitation (MR) post-cardiac implantable electronic devices (CIED).

Supplementary Figure 13: Contour-enhanced funnel plots of tricuspid regurgitation (TR) *(panels A to C),* and mitral regurgitation (MR) *(panels D to F)* post-cardiac implantable electronic devices (CIED).

**Methods**

***Search strategy:*** PubMed/MEDLINE, EMBASE, and Cochrane Library databases were systematically searched to identify all relevant English language studies restricted to human adults published from inception until October 31^st^, 2023. The search terms used were as follows: : **“(tricuspid regurgitation OR mitral regurgitation) AND (pacemaker OR pacing permanent pacemaker OR right ventricular pacing OR implantable cardioverter defibrillator OR ICD OR cardiac resynchronization therapy defibrillator OR CRTD OR cardiac resynchronization therapy pacemaker OR CRTP OR biventricular pacing OR conduction system pacing OR His bundle pacing OR physiologic pacing OR left bundle branch pacing OR leadless pacemaker OR cardiac implantable electronic devices***)”.* Manual searches of bibliography of published articles were also undertaken.

**Supplementary Figure 1: Consort Diagram Tricuspid and Mitral Regurgitation post-cardiac implantable electronic devices.**

MANUAL SEARCHES

PUBMED/

MEDLINE

**N = 146**

COCHRANE LIBRARY

EMBASE

**N = 9160**

**N = 3123**

**N = 6**

**N = 12435**

/*

**IDENTIFICATION**

Non-human & non-English studies excluded**: N = 1434**

Conference abstracts**: N = 2056**

Commentaries, editorials, letters: **N = 823**

Reviews**: N = 1380**

Case reports**: N = 2726**

Duplicates after exclusion of above**: N = 1767**

**N = 11001**

**SCREENING**

**N = 2249**

Titles & abstracts screened

Unrelated or indeterminate design or irrelevant titles & abstracts, including tricuspid or mitral valve repair or replacement, and MitraClip**:** **N = 1895**

**N = 354**

Full text articles reviewed

**ELIGIBILITY**

Failure to fulfil inclusion and exclusion criteria or poor quality studies**:** **N =224**

**Tricuspid Regurgitation: N = 57 studies** retained for systematic review and meta-analysis reporting incidence of tricuspid regurgitation post-cardiac implantable electronic device implantation *-17 studies reported both tricuspid and mitral regurgitation*

**Mitral Regurgitation: N = 90 studies** retained for systematic review and meta-analysis reporting mitral regurgitation post-cardiac implantable electronic device implantation *– 17 studies reported both tricuspid and mitral regurgitation*

**INCLUDED**

**Supplementary Table 1: Baseline characteristic of studies comparing tricuspid regurgitation pre- and post-cardiac implantable electronic devices**.

| **Study** | **Year** | **Sample size** | **Study design** | **Mean Age (years)** | **Men %** | ***CIED Type*** | **PPM**  **n** | **ICD**  **n** | **CRT**  **n** | **LVEF %** | **CAD %** | **AF %** | **HF**  **%** | **HTN %** | **DM** | **CVA %** | **CKD %** | **COPD %** | **NOS** |
| --- | --- | --- | --- | --- | --- | --- | --- | --- | --- | --- | --- | --- | --- | --- | --- | --- | --- | --- | --- |
| Abu Sham’a et al (1) | 2013 | 193 | Prospective cohort | 69 | 86 | CRT | - | - | 193 | 24 | 71 | 24 | 100 | - | - | - | - | - | 9 |
| Addetia et al (2) | 2014 | 100 | Prospective cohort | 67 | 49 | RVP PPM/ICD/CRT | 20 | 53 | 27 | 35 | - | - | - | - | - | - | - | - | 8 |
| Al-bawardy et al (3) | 2015 | 1596 | Retrospective cohort | 60 | 61 | RVP PPM/ICD/CRT | 310 | 985 | 334 | 38 | 42 | - | - | - | - | - | - | - | 9 |
| Alizadeh et al (4) | 2011 | 115 | Prospective cohort | 66.6 | 49 | RVP PPM | 115 | - | - | 55.4 | - | - | - | - | - | - | - | - | 8 |
| Anvardeen et al (5) | 2019 | 128 | Prospective cohort | 66.8 | 27 | RVP PPM | 128 | - | - | - | - | - | - | - | - | - | - |  | 7 |
| Arabi et al (6) | 2015 | 41 | Prospective cohort | 63.6 | 75.6 | RVP PPM/ICD/CRT | 7 | 9 | 9 | 34.2 | - | - | - | 61 | 36.6 | - | - | 7.3 | 8 |
| Arps et al (7) | 2023 | 54 | Retrospective cohort | 70.1 | 55.6 | LLPPM | 54 | - | - | 52.3 | 38.9 | 64.8 | 22.2 | 74.1 | 27.8 | 16.7 | 35.2 | - | 7 |
| Baquero et al (8) | 2013 | 148 | Prospective cohort | 68 | 66.9 | RVP PPM/ICD/CRT | 53 | 59 | 36 | 40 | - | - | - |  | - | - | - | - | 9 |
| Beurskens et al LLPPM (9) | 2019 | 53 | Prospective cohort | 80 | 70 | LLPPM | 53 | - | - | 53.5 | 6 | - | 9 | 32 | 11 | 4 | 8 | - | 9 |
| Beurskens et al TVPPM (9) | 2019 | 53 | Prospective cohort | 79 | 70 | RVP PPM | 53 | - | - | - | 4 | - | 6 | 54 | 25 | 6 | 6 | - | 9 |
| Breeman et al (10) | 2023 | 87 | Retrospective cohort | 58.4 | 70 | RVP PPM | - | 87 | - | 37 | 36 | 28 | 100 | 43 | 14 | - |  | - | 8 |
| Chen et al CRT (11) | 2022 | 51 | Prospective cohort | 64.4 | 58.8 | CRT | 16 | 35 | 51 | 28.4 | - | 5.9 | 100 | 31.4 | 19.6 | - | 5.9 | - | 8 |
| Chen et al LBBP (11) | 2022 | 49 | Prospective cohort | 67.1 | 50 | LBBP | 25 | 25 | 49 | 29.1 | - | 8.2 | 100 | 28.8 | 24.5 | - | 8.2 |  | 8 |
| Cho et al (12) | 2019 | 530 | Retrospective cohort | 63.4 | 48.8 | RVP PPM | 530 | - | - | - | - | - | - | - |  | - | - | - | 9 |
| Chodor-Rozwadowska (13) | 2023 | 101 | Prospective cohort | 69 | 56.4 | RVP PPM/ICD/CRT | 47 | 28 | 16 | 50 | 51.5 | 36.6 | 40.6 |  | 28.7 | - | - | - | 7 |
| Dabas et al (14) | 2021 | 42 | Retrospective cohort | 74 | 45 | LLPPM | 42 | - | - | - | - | - | - | - | - | - | - | - | 7 |
| Delling et al (15) | 2016 | 634 | Retrospective cohort | 80 | 54 | RVP PPM | 634 |  | - | 48 | 41 | - | - | 34 | 28 | - | - | - | 9 |
| Fanari et al (16) | 2015 | 206 | Retrospective cohort | 74 | 56 | RVP PPM/ICD | 120 | 86 | - | 55 | - | - | - | - | - | - | - | - | 9 |
| Grieco et al HBP (17) | 2021 | 42 | Prospective cohort | 75.1 | 67 | HBP | 42 | - | - | 52.7 | 21 | 10 | - | 71 | 10 |  | 17 | - | 8 |
| Grieco et al RVP (17) | 2021 | 42 | Prospective cohort | 75.2 | 62 | RVP PPM | 42 | - |  | 54.6 | 19 | 12 | - | 64 | 10 | - | 21 | - | 8 |
| Grupper et al (18) | 2015 | 689 | Retrospective cohort | - | - | CRT | - | - | 689 | - | - | - | - |  | - | - | - | 15 | 9 |
| HU et al (19) | 2022 | 91 | Prospective cohort | 67 | 42.9 | LBBP | 91 | - | - | 63 | 28.6 | 5.5 |  | 56 | 209 | 13.2 | - | - | 7 |
| Haeberlin et al (20) | 2022 | 69 | Retrospective cohort | 78 | 74 | LLPPM | 69 | - | - | 60 |  | 36 |  | 81 | 29 | - | 55 |  | 8 |
| Hai et al (21) | 2021 | 64 | Retrospective cohort | 81.4 | 48.4 | LLPPM | 64 | - | - | 61.4 | 28.1 | 71.9 | 20.3 | 81.3 | 26.6 | - | 51.6 | 4.7 | 8 |
| Hasumi et al (22) | 2018 | 45 | Prospective cohort | - | - | HBP | - | - | - | - | - | - | - | - | - |  | - | 12.5 | 7 |
| Hemayat et al (23)RVA | 2014 | 82 | Prospective cohort | 63.9 | 37.8 | HBP | 82 | - | - | - | - | - | - | - | - |  | - | - | 8 |
| Hemayat et al RVOT (23) | 2014 | 82 | Prospective cohort | 58.5 | 37.8 | RVP PPM | 82 | - | - | - | - | - | - | - | - |  | - | - | 8 |
| Hoke et al (24) | 2014 | 239 | Retrospective cohort | 60 | 77 | RVP PPM/ICD | 48 | 191 |  | 39 | 64 | 31 | 191 |  | 18 |  |  | - | 9 |
| Jin et al LBBP (25) | 2022 | 46 | Prospective cohort | 75.5 | 52.2 | LBBP | 46 | - | - | 53 | 15.2 | 100 | 100 | 69.6 | 26.1 | 13 | 4.3 | - | 8 |
| Jin et al LVSP (25) | 2022 | 10 | Prospective cohort | 74.6 | 60 | LVSP | 10 | - | - | 53.2 | 10.1 | 100 | 100 | 50 | 20 | 20 | 10 |  | 8 |
| Kanawati et al (26) | 2021 | 165 | Prospective cohort | 76.5 | 66 | RVP PPM/ICD | 116 | 49 | - | - | 40.6 | 38.8 | 43 | 60 | 40 | - | - | 21.2 | 8 |
| Kim et al (27) | 2008 | 248 | Prospective cohort | 75.4 | 58.9 | RVP PPM/ICD | 174 | 74 |  | - | - | - | - | - | - | - | - | - | 9 |
| Klustein et al (28) | 2009 | 410 | Retrospective cohort |  |  | RVP PPM | 410 | - | - | - | - | - | - | - | - | - | - | 9.4 | 9 |
| Kucukarslan et al (29) | 2006 | 61 | Prospective cohort | 53 | 72.1 | RVP PPM/ICD | 55 | 6 | - | 45 | 77 | - | - | - | - | - | - | - | 8 |
| Lee RC et al (30) | 2015 | 382 | Retrospective cohort | 68.2 | 66.6 | RVP PPM/ICD | 203 | 179 | - | 49.5 | 63.4 | 40.6 | 45 | 70.4 | 33.5 |  | 19.4 | 14.9 | 9 |
| Lee W-C et al (31) | 2021 | 1075 | Retrospective cohort | 72 | 48.4 | RVP PPM | 1075 | - | - | 69 | 18.2 | 30.04 |  | 72 | 34.1 | 15.7 | 18.2 |  | 9 |
| Leibowitz et al(32) | 2000 | 35 | Prospective cohort | 67 | 85.7 | RVP PPM/ICD | 20 | 25 | - | - | 57.1 | - | - | - | - | - | - | - | 7 |
| Livesay et al (33) | 2022 | 154 | Retrospective cohort | 71.8 | 54.14 | RVP PPM/ICD/CRT | 99 | 18 | 37 | - | - | - | - | - | - | - | - | - | 8 |
| Mao et al RVP (34) | 2023 | 33 | Prospective cohort | 72.9 | 63.6 | RVP | 33 | 0 | 0 | 67.8 | 30.3 | 39.4 | - | 84.8 | 27.3 | - | - | - | 8 |
| Mao et al LBBP (34) | 2023 | 45 | Prospective cohort | 72.7 | 55.6 | LBBP | 45 | 0 | 0 | 55.2 | 37.8 | 37.8 | - | 77.8 | 37.8 | - | - | - | 8 |
| Marincheva et al (35) | 2022 | 111 | Prospective cohort | 74.4 | 58.6 | RVP PPM/ICD | 90 | 21 |  | 53.1 | 44.1 | 44.1 | 21.6 | 86.5 | 44.8 |  | 27 | 8.1 | 8 |
| Markman et al CRT (36) | 2020 | 105 | Retrospective cohort | 64 | 66 | CRT | - | - | 105 | 30.1 |  | 38 | 66 | 67 | - | - | - | - | 9 |
| Markman et al non-CRT (36) | 2020 | 478 | Retrospective cohort | 63.8 | 60 | RVP PPM/ICD | 372 | 105 | - | 52.4 | - | 43 | 38 | 64 | - | - | - | - | 9 |
| Nakajima et al (37) | 2020 | 143 | Retrospective cohort | 62 | 65.7 | RVP PPM/ICD/CRT | 52 | 41 | 50 | - | 12.6 | 30.8 | - | - | - | - | - | - | 8 |
| Papageorgiou et al (38) | 2020 | 304 | Retrospective cohort | 58 | 41.1 | RVP PPM/ICD/CRT | 95 | 203 | 6 | - | - | - | - | - | - | - | - | - | 9 |
| Riesenhuber et al (39) | 2021 | 990 | Retrospective cohort | 70.4 | 61.9 | RVP PPM | 990 | - | - | - | 57.3 | 55.9 | 52.5 | - | - | - | - | - | 9 |
| Rothschild et al (40) | 2017 | 36 | Prospective cohort | 78 | 60 | - |  | - | - | - | - | - |  | - | - | - | - | - | 7 |
| Sadreddini et al (41) | 2014 | 89 | Retrospective cohort | 69.6 | 34.8 | RVP PPM/ICDCRT | 26 | 37 | 25 | 37.9 | 60.7 | - | 47.2 | - | - | - | 23.6 | - | 9 |
| Saito et al (42) | 2015 | 145 | RCT | 75 | 67 | RVP PPM | 145 | - | - | 57 | 26 | 20 | - | 58 | 21 | 4 | - | - | 9 |
| Salaun et al (43) | 2018 | 23 | Prospective cohort | 85.2 | 48 | LLPPM | 23 | - | - | 61.7 | 26 | 9 | - | 61 | 17 |  | 52 | 9 | 7 |
| Schleifer et al (44) | 2018 | 94 | RCT | 74.7 | 66.7 | RVP PPM/ICD | 52 | 42 | - | 62.5 | - | - | - | - | - | - | - | - | 9 |
| Seo J et al (45) | 2020 | 429 | Retrospective cohort | 67 | 41 | RVP PPM | 429 | - | - | 65.6 | 12.4 | 30.5 | - | 59.7 | 24.2 | - | - | - | 9 |
| Seo Y et al (46) | 2020 | 373 | Retrospective cohort | 63.1 | 65.4 | RVP PPM/ICD | 115 | 99 | 93 | - | - | - | - | - | - | - | - | 11 | 8 |
| Shan et al (47) | 2018 | 16 | Prospective cohort | 70.6 | 68.8 | HBP | - | - | - | 35.7 | - | - | 100 | - | - | - | - | - | 7 |
| Stassen et al (48) | 2022 | 852 | Retrospective cohort | 65.2 | 76.5 | CRT | - | - | 852 | - | 58.8 | - | 100 | 47.8 | 21 | - | - | - | 9 |
| Vaidya et al LLPPM (49) | 2019 | 86 | Prospective cohort | 80.5 | 63 | LLPPM | 90 | - | - | 59 | 46 | 86 | 46 | 73 | 22 | 12 | - | - | 9 |
| Vaidya et al TVPPM (49) | 2019 | 89 | Prospective cohort | 78.2 | 63 | RVP PPM | 90 |  |  | 60 | 47 | 90 | 44 | 42 | 24 | 18 | - | - | 9 |
| Van De Heyning et al (50) | 2019 | 328 | Prospective cohort | 69 | 62 | RVP PPM/ICD/CRT | 174 | 107 | 47 | 49 | 29 | - | - | 70 | 26 | - | - | - | 9 |
| Vaturi et al (51) | 2010 | 23 | Prospective cohort | 78 | 56.5 | - | - | - | - | - | - | - | - | - | - | - | - | - | 7 |
| Wang et al (52) | 2023 | 8 | Prospective cohort | 74 | 75 | HBP | 8 | 0 | 0 | 62 | 50 | 100 |  | 37.5 | 25 | 25 | 37.5 |  | 7 |
| Webster et al (53) | 2007 | 123 | Retrospective | 16 |  | - | - | - | - | - | - | - | - | - | - | - | - | - | 8 |
| Wiechecka et al(54) | 2020 | 110 | Prospective cohort | 75.1 | 56 | RVP PPM/ICD/CRT | 87 | 21 | 2 | - | 37 | 56 | 45 | 77 | 26 | - | - | - | 8 |
| Wu et al BIV (55) | 2021 | 54 | Prospective cohort | 68.3 | 53.7 | CRT | 0 | 44 | 54 | 29.7 | 13 | 20.4 | 100 | 50 | 29.6 | - | 24.1 | - | 9 |
| Wu et al HBP (55) | 2021 | 49 | Prospective cohort | 68.3 | 63.3 | HBP | 1 | 35 | 48 | 31 | 16.3 | 32.7 | 100 | 40.8 | 12.2 | - | 20.4 | - | 9 |
| Wu et al LBBP (55) | 2021 | 32 | Prospective cohort | 67.2 | 43.8 | LBBP | 4 | 21 | 28 | 30.4 | 3.1 | 21.9 | 100 | 50 | 37.5 | - | 15.6 | - | 9 |
| Yu et al (56) | 2020 | 284 | Retrospective cohort | 72 | 53.2 | RVP PPM | 284 | - | - | 62.3 | 32.7 | 36.6 | - | 72.9 | 37.3 | - | - | - | 9 |
| Zhang et al (57) | 2023 | 29 | Prospective cohort | 68.3 | 48.3 | LBBP | - | - | - | 32.2 | 27.6 | 20.7 | 100 | - | - | - | - | - | 8 |

AF: atrial fibrillation; CAD; coronary artery disease CIED: cardiac implantable electronic device; CKD: chronic kidney disease; CRT: cardiac resynchronization therapy (with biventricular pacing leads); CVA: cerebrovascular accident; DM: diabetes mellitus; HBP: His bundle pacing; HTN: hypertension; ICD: implantable cardioverter defibrillator; ICM: ischemic cardiomyopathy; LBBB: left bundle branch block; LBBP: left bundle branch pacing; LLPPM: leadless permanent pacemaker; LVEF: left ventricular ejection fraction; LVSP: left ventricular septal pacing; NOS: Newcastle-Ottawa Scale (for quality assessment with 0-3=poor quality, 4-7=fair quality, 8-9=good quality); PPM: permanent pacemaker; RCT: randomized controlled trial; RVP: right ventricular pacing; TVPPM transvenous permanent pacemaker.

**Supplementary Table 2: Baseline characteristic of studies comparing mitral regurgitation pre- and post-cardiac implantable electronic devices**.

| **Author** | **Year** | **Sample size** | **Study design & Number of sites** | **Mean Age (years)** | **Men**  **%** | **CIED Type** | **LVEF %** | **CAD**  **%** | **AF %** | **HF %** | **HTN %** | **DM %** | **CVA %** | **CKD %** | **COPD**  **%** | **NYHA class II/IV % or mean** | **QRS**  **Duration (ms)** | **ACEI/ARB/ARNI %** | **BBs%** | **MRA %** | **Diuretics** | **NOS**  **Stars** |
| --- | --- | --- | --- | --- | --- | --- | --- | --- | --- | --- | --- | --- | --- | --- | --- | --- | --- | --- | --- | --- | --- | --- |
| Achilli et al (58) | 2006 | 133 | Prospective cohort | 72 | 75.0 | CRT | 25.0 | 47 | 14 | 100 | - | - | - | - | - | 75 | 157 | - | - | - | - | 8 |
| Achilli et al (59) | 2008 | 106 | Prospective cohort | 70 | 70.8 | CRT | 24.5 | 40 | - | 100 | - | - | - | - | - | 44 | 156 | - | - | - | - | 6 |
| Adelstein et al (60) | 2008 | 309 | Retrospective cohort | 66.6 | 76.4 | CRT | 22.1 | 60 | 45.3 | 100 | - |  | - | - | - | class 3.1 | 171.9 | 87.1 | 82.2 | 27.9 | - | 7 |
| Alizadeh et al (4) | 2011 | 115 | Prospective cohort | 66.6 | 49 | RVP PPM | 55.4 | - | 0 | - | - | - | - | - | - | - | - | - | - | - | - | 8 |
| António et al: <65 years (61) | 2010 | 51 | Prospective cohort | 54.7 | 66.7 | CRT | 23.9 | 33.3 | 12 | 100 | - | 13.7 | - | - | - | 82.2 | 141.2 | - | - | - | - | 7 |
| António et al:≥65 years (61) | 2010 | 36 | Prospective cohort | 71.8 | 58.3 | CRT | 24.4 | 38.9 | 19.4 | 100 | - | 29.4 | - | - | - | 97.1 | - | - | - | - | - | 8 |
| Atta et al QRS ≥130ms(62) | 2013 | 40 | Prospective observational | 52.4 | 77.5 | CRT | 26.6 | - | - | 100 | 47.5 | 70 | - | - | 15 | Class 3.2 | - | 92.5 | 87.5 | 67.5 | 82.5 | 7 |
| Bakker et al (63) | 2000 | 12 | Retrospective cohort | 64.4 | 40.0 | CRT | 15 | 33.3 | 0 | 100 | - | - | - | - | - | 66.7 | 194 | - | - | - | - | 6 |
| Beurskens et al, LLPPM (9) | 2019 | 53 | Prospective cohort | 80 | 70 | LLPPM | 53.5 | 6 | - | 9 | 32 | 11 | 4 | 8 | 4 | - | - | - | - | - | - | 9 |
| Beaudoin et al (64) | 2016 | 133 | Retrospective cohort | 65 | 78 | CRT | 25 | 45 | 36 | 100 | 63 | 32 | - | - | - | 90 | 160 | 78 | 83 | 29 | 77 | 8 |
| Binda et al (65) | 2018 | 172 | Prospective registry | 71 | 78 | CRT | 26 | 36 | 7 | 100 | 42 | 30 | - | - |  | 48 | 166 | 88 | 90 | - | - | 7 |
| Bordachar et al (66) | 2004 | 33 | Prospective cohort | 69 | 64 | CRT | 26 | 45 | - | 100 | - | - | - | - | - | 4.2±0.4 | 169 | 100 | 79 | 73 | 100 | 9 |
| Boriani et al (67) | 2012 | 659 | Prospective registry | 66.5 | 90 | CRT | 26 | 68 | 28.1 | 100 | 20.5 | 14.7 | - | 9.6 | 10.9 | 79.8 | 161 | 73 | 54 | - | 88 | 9 |
| Brandt et al (68) | 2006 | 20 | Prospective cohort | 68/61 | 80 | CRT | 24 | 25 | - | 100 | - | - | - | - | - | 100 | 150 | 100 | 90 | 80 | 100 | 8 |
| Breithardt et al (69) | 2003 | 24 | Prospective cohort | 63 | 79.2 | CRT | 21 | 50 | - | 100 | - | - | - | - | - | 96 | 176 | - | - | - | - | 8 |
| Brzezińska et al (70) | 2016 | 66 | Prospective cohort | 62/64 | 74 | CRT | 26/24 | 48.5 | 9.1 | 100 | 57.6 | 30.3 | - | - | - | 80.3 | 168/160 | 96.9 | 96.9 | 74 | 87.9 | 7 |
| Cabrera-Bueno et al (71) | 2010 | 76 | Prospective cohort | 63.4 | 71.1 | CRT | 22/20 | 25 | - | 100 | - | - | - | - | - | - | 169.4 | 97.3 | 88 | 72 | 100 | 8 |
| Chatterjee et al (72) | 2016 | 426 | Retrospective cohort | 66 | 66 | CRT | 28 | 59 | - | 100 | - | - | - | - | - | 97 | 151 | - | - | - | - | 9 |
| Chen et al, LBBP (11) | 2022 | 49 | Prospective cohort | 67.1 | 50 | LBBP | 29.1 | - | 8.2 | 100 | 28.6 | 24.5 | - | 8.2 | - | 91.8 | 180.2 | 98 | 98 | 93.9 | 93.9 | 8 |
| Chen et al, CRT (11) | 2022 | 51 | Prospective cohort | 64.4 | 58.8 | CRT | 28 | - | 5.9 | 100 | 31.4 | 19.6 | - | 5.9 | - | 88.2 | 175.7 | 98 | 100 | 98 | 90.2 | 8 |
| Chodor-Rozwadowska (13) | 2023 | 101 | Prospective cohort | 69 | 56.4 | RVP PPM/ICD | 50 | 51.5 | 36.6 | - | 28.7 | - | - | - | 6.9 | 7.92 | - | - | - | - | - | 7 |
| Cipriani et al (73) | 2016 | 1122 | Prospective cohort, multicenter | 66.4 | 78% | CRT | 27.8 | 47 | 31.7 | 100 | - | - | - | - | - | 71.8 | 152.9 | 82.4 | 77 | - | 90.3 | 8 |
| Cleland et al (74) | 2008 | 409 | RCT (CARE-HF) | 66.5 | 74.3% | CRT | 24.6 | 45.5 | - | 100 | - | - | - | - | - | I5.6 | 160 | 95 | 72.9 | 54 | 42.8 | 9 |
| Delnoy et al SR (75) | 2007 | 167 | Prospective registry | 72 | 68.3% | CRT | 22 | 41.3 | 0 | 100 | - | - | - | - | - | 78.8% | 171 | - | - | - | - | 8 |
| Delnoy et al AF (75) | 2007 | 96 | Prospective registry | 73 | 75.0 | CRT | 25 | 32.3 | 100 | 100 | - | - | - | - | - | 76.9 | 171 | - | - | - | - | 8 |
| Di Biase et al (76) | 2011 | 794 | Prospective cohort, multicenter | 66 | 75 | CRT | 24 | 50 | 44 | 100 | - | - | - | - | - | Class 2.99 | 162 | - | - | - | - | 8 |
| Donnellan et al RVP (77) | 2019 | 53 | Retrospective cohort | 76.5 | 47 | RVP PPM | - | - | 71.7 | - | - | - | - | - | - | - | - | - | - | - | - | 8 |
| Donnellan et al CRT (77) | 2019 | 25 | Retrospective cohort | 75 | 92 | CRT | 25 | - | 13 | - | - | - | - | - | - | 74 | - | - | - | - | - | 8 |
| Ennezat et al (78) | 2006 | 21 | Prospective cohort | - | - | CRT | 22 | - | - | 100 | - | - | - | - | - | - | - | - | - | - | - | 7 |
| Fanari et al (16) | 2015 | 206 | Retrospective cohort | 74 | 56 | RVP PPM/ICD | 55 | - | - | - | - | - | - | - | - | - | - | - | - | - | - | 9 |
| Fukuda et al (79) | 2005 | 19 | Prospective cohort | 64 | 78.9 | CRT | 23.7 | - | - | 100 | - | - | - | - | - | - | 161 | - | - | - | - | 7 |
| Gras et al (80) | 2002 | 46 | Prospective cohort | 66 | 76 | CRT | 22 | 43.5 | - | 100 | - | - | - | - | - | 100 | 178 | - | - | - | - | 9 |
| Haeberlin et al (20) | 2022 | 69 | Retrospective cohort | 78 | 74 | RVP LLPPM | 60 | 46 | 36 | - | 81 | 29 | - | 55 | - | - | - | - | 57 | - | - | 8 |
| Hai et al (21) | 2021 | 64 | Retrospective cohort | 81.4 | 48.4 | RVP LLPPM | 61.4 | 28.1 | 71.9 | 20.3 | 81.3 | 28.1 | - | 51.6 | 4.7 | - | - | - | - | - | - | 8 |
| Hemayat et al RVA (23) | 2014 | 82 | Prospective cohort | 63.9 | 37.8 | RVP PPM | - | - | - | - | - | - | - | - | - | - | - | - | - | - | - | 8 |
| Hemayat et al RVOT (23) | 2014 | 82 | Prospective cohort | 58.5 | 37.8 | RVP PPM | - | - | - | - | - | - | - | - | - | - | - | - | - | - | - | 8 |
| Iori et al (81) | 2014 | 42 | Retrospective cohort | 66.3 | 78.6 | CRT | 21 | **35.75** | - | **100** | **-** | **-** | - | - | - | - | - | - | - | - | - | 7 |
| Jin et al (82) | 2018 | 296 | Prospective cohort | 59 | 68.2 | CRT | 29 | **21.3** | 14.5 | **100** | **-** | **-** | - | - | - | 75.4 | 160.8 | 78 | 89.5 | 92.6 | - | 8 |
| Jin et al, LBBP (25) | 2022 | 46 | Prospective cohort | 75.5 | 52.2 | LBBP | 53 | **15.2** | - | **100** | **69.6** | **26.1** | 13 | 4.3 | - | - | - | - | - | - | - | 8 |
| Jin et al, LVSP (25) | 2022 | 10 | Prospective cohort | 74.6 | 60 | LVSP | 53.2 | **10.1** | - | **100** | **50** | **20** | 20 | 10 | - | - | - | - | - | - | - | 8 |
| Kanzaki et al (83) | 2004 | 26 | Prospective cohort | - | - | CRT | 24 | **-** | - | **100** | **-** | **-** | - | - | - | - | 168 | - | - | - | - | 8 |
| Karaca et al (84) | 2018 | 95 | Prospective cohort | 64.1 | 64 | CRT | 26.6 | **58** | 36 | **100** | **72** | **33** | - | - | - | 67 | 159.9 | 95 | 94 | 77 | 93 | 8 |
| Karvounis et al (85) | 2006 | 22 | Prospective cohort | 65 | 72.7 | CRT | 18 | **59** | - | **100** | **-** | **-** | - | - | - | 68.15 | 165 | - | - | - | - | 7 |
| Kelarijani et al (86) | 2008 | 60 | Prospective cohort | 59 | 76.7 | CRT | 21% | **46.7** | - | **100** | **-** | **-** | - | - | - | 100 | 144 | - | - | - | - | 8 |
| Killu et al: ≤80 years (87) | 2013 | 638 | Prospective cohort | 66.5 | 77 | CRT | 23.5 | **54.7** | 45.3 | **100** | **-** | **-** | - | - | - | class 3 | 164.5 | 82.5 | 86.5 | - | - | 9 |
| Killu et al: >80 years (87) | 2013 | 90 | Prospective cohort | 83.7 | 71 | CRT | 24.3 | **70** | 30 | **100** | **-** | **-** | - | - | - | class 3 | 171.5 | 75.9 | 90.7 | - | - | 9 |
| Kranig et al (88) | 2015 | 39 | RCT | 68 | 85 | CRT | 27 | **67** | - | **100** | **-** | **-** | - | - | - | 95 | 171 | 90 | 77 | 59 | 82 | 9 |
| Kuppahally et al (89) | 2009 | 35 | Prospective cohort | 55/62 | 77/92 | CRT | 25.7/28.0 | **91/38** | - | **100** | **-** | **-** | - | - | - | 100 | 164/156 | - | - | - | - | 8 |
| Lancellotti et al (90) | 2004 | 27 | Prospective cohort | - | - | CRT | 29 | **-** | - | **100** | **-** | **-** | - | - | - | - | - | - | - | - | - | 6 |
| Lau et al (91) | 2000 | 11 | Prospective cohort | 61 | 63.6 | CRT | 21.6 | **-** | - | **100** | **-** | **-** | - | - | - | 100 | 165 | 81.8 | 54.5 | - | 100 | 7 |
| Liang et al (92) | 2010 | 83 | Prospective cohort | 65 | 75.7 | CRT | 28 | **47** | 5 | **100** | **38** | **10** | - | - | - | 100 | - | 85.4 | 79.3 | 29.3 | 76.8 | 8 |
| Linde et al SR (93) | 2002 | 67 | MUSTIC study | 63 | 75 | CRT | 22.5 | **37** | - | **100** | **-** | **-** | - | - | - | 100 | 176 | 96 | 28 | 22 | 94 | 9 |
| Linde et al AF(93) | 2002 | 64 | MUSTIC study | 65 | 81 | CRT | 26.7 | **27** | - | **100** | **-** | **-** | - | - | - | 100 | 206 | 100 | 25 | 15 | 98 | 9 |
| Madaric et al (94) | 2007 | 28 | Prospective cohort | 67 | 82 | CRT | 25 | **50** | - | **100** | **-** | - | - | - | - | Class 3.0 | 171 | 100 | 79 | 82 | 79 | 8 |
| Mangiavacchi et al (95) | 2006 | 156 | Prospective cohort | 65.6 | 74.4 | CRT | 31.4 | **48.7** | - | **100** | **-** | - | - | - | - | 83.2 | 171.7 | 93.9 | 75 | 62.2 | 79.4 | 9 |
| Marechaux et al (96) | 2009 | 26 | Prospective cohort | 65 | 76 | CRT | 23% | **37** | - | **100** | **-** | - | - | - | - | - | - | 100 | 88 | 49 | 78 | 8 |
| Marincheva et al (35) | 2022 | 111 | Prospective cohort | 74.4 | 58.6 | RVP PPM/ICD | 53.1 | **44.1** | 44.1 | **100** | **86.5** | 46.8 | - | 27 | 8.1 | - | - | 77.5 | 64 | 14.4 | 22.5 | 8 |
| Markman et al CRT (36) | 2020 | 105 | Retrospective cohort, | 64 | 66 | CRT | 30.1 | **-** | - | **-** | **-** | - | - | - | - | - | 152 | - | - | - | - | 9 |
| Markman et al non-CRT (36) | 2020 | 478 | Retrospective cohort | 63.8 | 60 | RVP PPM /ICD | 52.4 | **-** | - | **100** | **-** | - | - | - | - | - | 116 | - | - | - | - | 9 |
| Martens et al (97) | 2018 | 31 | Prospective cohort | 66 | 74 | CRT | 29 | **40** | - | **100** | **71** | 23 | - | - | 13 | 100 | 149 | 90 | 87 | 71 | - | 9 |
| Matsumoto K (98) | 2011 | 44 | Prospective cohort | 77 | 70.5 | CRT | 28 | **-** | - | **100** | **-** | - | - | - | - | 100 | 160.5 | 86.3 | 88.6 | 63.6 | 88.6 | 8 |
| Menardi et al (99) | 2008 | 100 | Prospective cohort | 70 | - | CRT | 19.6 | **32** | - | **100** | **4** | - | - | - | - | 95 | - | - |  | - | - | 9 |
| Mihos et al (100) | 2017 | 71 | Retrospective cohort | 67 | 78 | CRT | 25 | **52** | 4 | **100** | **73** | 30 | - | - | - | Class 3.0 | 160 | 75 | 93 | 34 | 79 | 9 |
| Molhoek et al ICM(101) | 2004 | 34 | Prospective cohort | 65 | 67.5 | CRT | 21 | **100** | - | **100** | **-** | - | - | - | - | Class 3.1 | 175 | - | - | - | - | 7 |
| Molhoek et al NICM (101) | 2004 | 40 | Prospective cohort | 64 | 88.2 | CRT | 23 | **0** | - | **100** | **-** | - | - | - | - | Class 3.2 | 178 | - | - | - | - | 7 |
| Naqvi et al: Improved MR (102) | 2008 | 18 | Prospective cohort | 71 | 56 | CRT | 30.3 | **44** | 44 | **100** | **60** | 29 | - | - | - | Class 3.2 | - | 78 | 83 | 33 | 78 | 9 |
| Naqvi et al: Unimproved MR (102) | 2008 | 17 | Prospective cohort | 59 | 59 | CRT | 27.1 | **65** | 41 | **100** | **44** | 33 | - | - | - | Class 3.1 | - | 59 | 59 | 35 | 94 | 9 |
| Onishi et al (103) | 2013 | 240 | Prospective cohort | 65 | 71 | CRT | 24 | **54** | 17 | **100** | **-** | - | - | - | - | 11 | 159 | - | - | - | - | 8 |
| Pfau et al (104) | 2010 | 215 | Retrospective cohort | 62.8 | 73.4 | CRT | 22.2 | **44.2** | 22.8 | **100** | **60** | - | - | - | - | 88 | - | 96.7 | 82.4 | 26.5 | 88.8 | 8 |
| Porciani et al (105) | 2006 | 30 | Prospective cohort | 73.7 | 93.3 | CRT | 26/28 | **46.7** | - | **100** | **-** | - | - | - | - | 100 | 140 | 82 | 78 | 15 | 80 | 8 |
| Praus et al (106) | 2012 | 58 | Prospective cohort | 67 | - | CRT | 22 | **57** | - | **100** | **-** | - | - | - | - | 70.7 | 193 | - | - | - | - | 8 |
| Rao et al (107) | 2007 | 306 | RCT | 66.7 | 67.0 | CRT | 28.1 | **63.7** | - | **100** | **-** | - | - | - | - | 100 | 166.7 | 87.1 | 81.2 | 38.2 | 86.3 | 9 |
| Reuter et al (108) | 2000 | 47 | Prospective cohort | 64 | 81 | CRT | 23 | **45** | _ | **100** | **-** | - | - | - | - | 94 | 193 | - | - | - | - | 8 |
| Rocha et al (109) | 2015 | 116 | Prospective cohort | 64.8 | 69.8 | CRT | 29 | **29.3** | _ | **100** | **-** | - | - | - | - | 100 | 160 | 97.4 | 88.7 | - | 31.9 | 9 |
| Sadreddini et al (41) | 2014 | 89 | Retrospective cohort | 69.6 | 34.8 | RVP PPM/ICD | 37.9 | **60.7** | - | **47.2** | **71.6** | 19.1 | - | 23.6 | - | - | - | 75.3 | 86.5 | 39.3 | 53.9 | 9 |
| Salaun et al (43) | 2018 | 23 | Prospective cohort | 85.2 | 48 | RVP LLPPM | 61.7 | **26** | 9 | - | 61 | 17 |  | 52 | 9 | 22 | - | 52 | 22 | - | - | 7 |
| Sassone et al (110) | 2001 | 27 | Prospective cohort | 71 | 51.9 | RVP PPM | - | **-** | - | **-** | **-** | - | - | - | - | - | - | - | - | - | - | 7 |
| Sénéchal et al (111) | 2010 | 57 | Prospective cohort | 71 | 75 | CRT | 22 | **-** | - | **100** | **-** | - | - | - | - | 100 | 162 | 95 | 86 | 54 | 95 | 9 |
| Shan et al (47) | 2018 | 16 | Prospective cohort | 70.6 | 68.8 | HBP | 35.7 | **-** | - | **-** | **-** | - | - | - | - | - | - | - | - | - | - | 7 |
| Sitges et al (112) | 2009 | 57 | Prospective cohort | 69 | 77 | CRT | 23 | **40** | - | **100** |  | - | - | - | - | 17% | - | - | - | - | - | 9 |
| Solis et al (113) | 2009 | 34 | Prospective cohort | 66 | 68 | CRT | 19 | **47** | - | **100** | **-** | - | - | - | - | 100 | - | - | - | - | - | 9 |
| Solomon et al (114) | 2010 | 749 | RCT (MADIT-CRT) | 64.4 | 75.4 | CRT | 29 | **54.9** | - | **100** | **63,4** | 29.0 | - | - | - | 85.4 | 158.6 | 95.1 | 93.9 | 31.9 | 76.2 | 9 |
| Stellbrink et al (115) | 2001 | 25 | Prospective cohort (PATH-CHF) | 60 | 52 | CRT | 22 | **25** | - | **100** | **-** | - | - | - | - | Class 3.0 | - | - | - | - | - | 8 |
| St John Sutton et al (116) | 2006 | 176 | RCT (MIRACLE Study) | 63.9 | 68.4 | CRT | 24 | **46.6** | - | **100** | **-** | - | - | - | - | 90.4 | 167.1 | 93 | 62 | - | 93 | 9 |
| St John Sutton et al (117) | 2009 | 419 | RCT (REVERSE Study) | 62.9 | 78 | CRT | 27.2 | **56** | - | **100** | **-** | 22 | - | - | - | 82 | 153 | 96 | 96 | - | 81 | 9 |
| Stockburger et al (118) | 2008 | 21 | Prospective cohort | 65 | 81 | CRT | 23 | **38** | - | **100** | **-** | - | - | - | - | - | 165 | 100 | 95 | 66.7 | 90.5 | 9 |
| Stolfo et al (119) | 2015 | 44 | Retrospective cohort | 58 | 61.4 | CRT | 24 | **-** | 21.4 | **100** | **-** | - | - | - | - | 44.4 | 164 | 93 | 87 | 57.1 | 77 | 9 |
| Tournoux et al (120) | 2007 | 53 | Prospective cohort | 68.8 | 69 | CRT | 21.9 | **69.8** | 24.5 | **100** | **-** | - | - | - | - | 100 | 169 | 49 | 75.5 | 37.7 | 88.75 | 9 |
| Upadhyay et al (121) | 2015 | 439 | Prospective cohort | 70.1 | 79.5 | CRT | 25% | **58.1** | 48 | **100`** | **73.8** | 39.9 | - | 6.7 | - | Class 2.97 | 162 | 80 | 90.4 | 34.4 | 85.4 | 9 |
| Upadhyay et al (122) | 2021 | 30 | Retrospective cohort | 68 | 73 | HBP | 31 | **26.6** | 33 | **-** | **77** | 33 | - | 67 | - | 63 | 162 |  |  |  |  | 8 |
| van Bommel et al (123) | 2011 | 98 | Prospective cohort | 71 | 72 | CRT | 23 | **63** | - | **100** | **-** | 18 | - | - | - | Class 3.2 | 166 | 88 | 58 | 50% | 88 | 9 |
| van der Bijl et al (124) | 2019 | 1313 | Prospective cohort | 66 | 77 | CRT | 27 | **59** | 8.9 | **100** | **19** | - | - | - | - | - | 156 | 80.4 | 67.3 | 39.9 | 72.5 | 9 |
| Verhaert et al (125) | 2012 | 266 | Prospective cohort | 69 | 69 | CRT | 25 | **51** | - | **100** | **-** | - | - | - | - | 90 | 162 | 86 | 85 | - | - | 9 |
| Vinereanu et al (126) | 2007 | 22 | Prospective cohort | 63 | - | CRT | - | **-** | - | **100** | **-** | - | - | - | - | - | - | - | - | - | - | 7 |
| Wang et al (52) | 2023 | 8 | Prospective cohort | 74 | 75 | HBP | 62 | **50** | 100 | **-** | **37.5** | **25** | - | 37.5 | - | - | - | 50 | 62.5 | 25 | 87.5 | 7 |
| Witte et al (127) | 2008 | 11 | Prospective cohort | 59 | - | CRT | 25 | **54.5** | - | **100** | **-** | - | - | - | - | - | 172 | 100 | 100 | 54.5 | 83 | 8 |
| Wu et al BIV (55) | 2021 | 54 | Prospective cohort | 68.3 | 53.7 | CRT | 29.7 | **14** | 20.4 | **100** | **50** | 29.6 | - | 24.1 | - | 70.4 | 161.1 | 90.7 | 88.9 | 98.1 | 98.1 | 8 |
| Wu et al, HBP (55) | 2021 | 49 | Prospective cohort | 68.3 | 63.3 | HBP | 31 | **16.3** | 32.7 | **100** | **40.8** | **12.2** | - | 20.4 | - | 69.4 | 170.3 | 89.8 | 93.9 | 91.8 | 98 | 8 |
| Wu et al, LBBP (55) | 2021 | 32 | Prospective cohort | 67.2 | 43.8 | LBBP | 30.4 | **3.1** | 21.9 | **100** | **50** | 37.5 | - | 16.6 | - | - | 166.2 | 90.6 | 84.4 | 90.6 | 96.9 | 8 |
| Ypenburg et al (128) | 2008 | 68 | Prospective cohort | 68 | 70.5 | CRT | 23% | **57** | - | **100** | **-** | - | - | - | - | 92.6 | 159 | 90 | 76 | 40 | 93 | 9 |
| Yu et al (129) | 2006 | 51 | Prospective cohort | 66 | 72.5 | CRT | 25.2 | **43.1** | - | **100** | **-** | - | - | - | - | Class 3.34 | 163 | 94 | 71 | 37 | 98 | 9 |
| Yu et al (56) | 2020 | 284 | Retrospective cohort | 72 | 53.2 | RVP PPM | 62.3 | **32.7** | 36.6 | **10.9** | **72.9** | 37.3 | - | - | - | - | - | - | - | - | - | 8 |
| Zanon et al (130) | 2004 | 45 | Prospective cohort | 72 | 82.2 | CRT | 23.7 | **-** | - | **100** | **-** | - | - | - | - | 100 | >150 | - | - | - | - | 8 |
| Zhang et al (57) | 2023 | 29 | Prospective cohort | 68.3 | 48.3 | LBBP | 32.2 | **27.6** | - | **-** | **-** | - | - | - | - | 96.6 | 171.9 | 37.9 | - | 100 | 100 | 8 |

ACEI: angiotensin converting enzyme inhibitor; ARB: angiotensin receptor blocker; ARNI: angiotensin receptor-neprilysin inhibitor; CIED: cardiac implantable electronic device;  CRT: cardiac resynchronization therapy (with biventricular pacing leads); HBP: His bundle pacing; ICD: implantable cardioverter defibrillator; ICM: ischemic cardiomyopathy; LBBB: left bundle branch block; LBBP: left bundle branch pacing; LLPPM: Leadless permanent pacemaker; LVEF: left ventricular ejection fraction; LVSP: left ventricular septal pacing; MRA: Mineralocorticoid receptor antagonist; NYHA: New York Heart Association; NOS: Newcastle-Ottawa Scale (for quality assessment with 0-3=poor quality, 4-7=fair quality, 8-9=good quality); PPM: permanent pacemaker; RCT: randomized controlled trial; RVP: right ventricular pacing.

**Supplementary Table 3: Significant tricuspid regurgitation post- cardiac implantable electronic devices and duration of follow-up.**

| **Study** | **Year** | **Sample size** | **Study Design** | **PPM** | **ICD** | **CRT** | **Pre-CIED** | | **Post-CIED** | | **Pre-CIED** | | **Post-CIED** | | **DOF (months)** |
| --- | --- | --- | --- | --- | --- | --- | --- | --- | --- | --- | --- | --- | --- | --- | --- |
|  |  |  |  |  |  |  | ***No significant TR (n)*** | ***Significant TR (n)*** | ***No significant TR (n)*** | ***Significant TR (n)*** | ***Mean TR Grade*** | ***Standard Deviation TR Grade*** | ***Mean TR Grade*** | ***Standard deviation TR Grade*** |  |
| Abu Sham’a et al (1) | 2013 | 193 | Prospective cohort |  |  | 193 | 158 | 35 | 133 | 60 |  |  |  |  | 12 |
| Addetia et al (2) | 2014 | 100 | Prospective cohort | 20 | 53 | 27 | 75 | 25 | 49 | 51 |  |  |  |  | 45.6 |
| Al-bawardy et al (3) | 2015 | 1596 | Retrospective cohort | 310 | 985 | 334 | 1241 | 255 | 1229 | 367 |  |  |  |  | 48 |
| Alizadeh et al (4) | 2011 | 115 | Prospective cohort | 115 |  |  | 105 | 10 | 79 | 36 |  |  |  |  | 48.96 |
| Anvardeen et al (5) | 2019 | 128 | Prospective cohort | 128 |  |  | 128 | 0 | 90 | 38 |  |  |  |  | 12 |
| Arabi et al (6) | 2015 | 41 | Prospective cohort | 7 | 9 | 9 | 31 | 10 | 7 | 34 |  |  |  |  | 12 |
| Arps et al (7) | 2023 | 54 | Retrospective cohort | 54 |  |  | 40 | 14 | 42 | 12 |  |  |  |  | 8.9 |
| Baquero et al (8) | 2013 | 148 | Prospective cohort | 53 | 59 | 36 | 123 | 25 | 115 | 33 |  |  |  |  | 32 |
| Beurskens et al LLPPM (9) | 2019 | 53 | Prospective cohort | 53 |  |  | 46 | 7 | 39 | 14 |  |  |  |  | 12 |
| Beurskens et al TVPPM (9) | 2019 | 53 | Prospective cohort | 53 |  |  | 49 | 4 | 33 | 20 |  |  |  |  | 16 |
| Breeman et al (10) | 2023 | 87 | Retrospective cohort |  | 87 |  | 66 | 21 | 56 | 31 |  |  |  |  | 20 |
| Chen et al (11) | 2022 | 51 | Prospective cohort | 16 | 35 | 51 |  |  |  |  | 1.45 | 0.69 | 1.11 | 0.4 | 12 |
| Chen et al (11) | 2022 | 49 | Prospective cohort | 25 | 25 | 49 |  |  |  |  | 1.17 | 0.86 | 1 | 0.52 | 12 |
| Cho et al (12) | 2019 | 530 | Retrospective cohort | 530 |  |  | 530 | 0 | 453 | 77 |  |  |  |  | 91.2 |
| Chodor-Rozwadowska et al (13) | 2023 | 101 | Prospective cohort | 47 | 28 | 16 | 91 | 10 | 72 | 29 |  |  |  |  | 13 |
| Dabas et al (14) | 2021 | 42 | Retrospective cohort | 42 |  |  | 30 | 12 | 33 | 9 |  |  |  |  |  |
| Delling et al (15) | 2016 | 634 | Retrospective cohort | 634 |  |  | 621 | 13 | 533 | 101 |  |  |  |  | 15.6 |
| Fanari et al (16) | 2015 | 206 | Retrospective cohort | 120 | 86 |  | 164 | 42 | 137 | 69 |  |  |  |  | 29 |
| Grieco et al (17) | 2021 | 42 | Prospective cohort | 42 |  |  | 30 | 13 | 38 | 4 |  |  |  |  | 6 |
| Grieco et al (17) | 2021 | 42 | Prospective cohort | 42 |  |  | 32 | 10 | 29 | 13 |  |  |  |  | 6 |
| Grupper et al (18) | 2015 | 689 | Retrospective cohort |  |  | 689 | 447 | 242 | 585 | 104 |  |  |  |  | 15 |
| HU et al (19) | 2022 | 91 | Prospective cohort | 91 |  |  | 60 | 31 | 59 | 32 |  |  |  |  | 18 |
| Haeberlin et al (20) | 2022 | 69 | Retrospective cohort | 69 |  |  | 54 | 15 | 51 | 18 |  |  |  |  | 11.4 |
| Hai et al (21) | 2021 | 64 | Retrospective cohort | 64 |  |  | 44 | 20 | 39 | 25 |  |  |  |  | 14.7 |
| Hasumi et al (22) | 2018 | 45 | Prospective cohort | 45 |  |  | 43 | 2 | 45 | 0.5 |  |  |  |  | 12.5 |
| Hemayat et al RVA (23) | 2014 | 82 | Prospective cohort | 82 |  |  | 82 | 0 | 78 | 4 |  |  |  |  | 12 |
| Hemayat et al RVOT (23) | 2014 | 82 | Prospective cohort | 82 |  |  | 82 | 0 | 81 | 1 |  |  |  |  | 12 |
| Hoke et al (24) | 2014 | 239 | Retrospective cohort | 48 | 191 |  | 212 | 27 | 128 | 111 |  |  |  |  | 18 |
| Jin et al* (82) | 2022 | 46 | Prospective cohort | 46 |  |  |  |  |  |  | 1 | 1-2 | 1 | 1 - 2.2 | 12 |
| Jin et al* (82) | 2022 | 10 | Prospective cohort | 10 |  |  |  |  |  |  | 2 | 1-3 | 1 | 1-2 | 12 |
| Kanawati et al (26) | 2021 | 165 | Prospective cohort | 116 | 49 |  | 165 | 0.5 | 121 | 44 |  |  |  |  | 29 |
| Kim et al(27) | 2008 | 248 | Prospective cohort | 174 | 74 |  | 179 | 69 | 141 | 107 |  |  |  |  | 3 |
| Klustein et al (28) | 2009 | 410 | Retrospective cohort | 410 |  |  | 410 | 0 | 335 | 75 |  |  |  |  | 9.4 |
| Kucukarslan et al (29) | 2006 | 61 | Prospective cohort | 55 | 6 |  | 52 | 9 | 49 | 12 |  |  |  |  | 12 |
| Lee RC et al(30) | 2015 | 382 | Retrospective cohort | 203 | 179 |  | 382 | 0 | 270 | 112 |  |  |  |  | 18.9 |
| Lee W-C et al (31) | 2021 | 1075 | Retrospective cohort | 1075 |  |  | 1075 | 0 | 957 | 118 |  |  |  |  | 58.8 |
| Leibowitz et al (32) | 2000 | 35 | Prospective cohort | 20 | 25 |  | 25 | 10 | 28 | 7 |  |  |  |  | 0.04 |
| Livesay et al (33) | 2022 | 154 | Retrospective cohort | 99 | 18 | 37 | 132 | 22 | 98 | 56 |  |  |  |  | 12 |
| Mao et al (34) | 2023 | 33 | Prospective cohort | 33 | 0 | 0 | 24 | 2 | 23 | 3 |  |  |  |  | 12 |
| Mao et al (34) | 2023 | 45 | Prospective cohort | 45 | 0 | 0 | 25 | 5 | 25 | 5 |  |  |  |  | 12 |
| Marincheva et al (35) | 2022 | 111 | Prospective cohort | 90 | 21 |  | 110 | 1 | 107 | 4 |  |  |  |  | 6 |
| Markman et al CRT (36) | 2020 | 105 | Retrospective cohort |  |  | 105 | 86 | 19 | 90 | 15 |  |  |  |  | 8.3 |
| Markman et al non-CRT (36) | 2020 | 478 | Retrospective cohort | 372 | 105 |  | 391 | 87 | 372 | 106 |  |  |  |  | 8.3 |
| Nakajima et al (37) | 2020 | 143 | Retrospective cohort | 52 | 41 | 50 | 143 | 0.5 | 114 | 29 |  |  |  |  | 10 |
| Papageorgiou et al (38) | 2020 | 304 | Retrospective cohort | 95 | 203 | 6 | 304 | 0.5 | 238 | 66 |  |  |  |  | 138.2 |
| Riesenhuber et al (39) | 2021 | 990 | Retrospective cohort | 990 |  |  | 727 | 263 | 551 | 439 |  |  |  |  | 5 |
| Rothschild et al (40) | 2017 | 36 | Prospective cohort |  |  |  | 36 | 0 | 30 | 6 |  |  |  |  |  |
| Sadreddini et al (41) | 2014 | 89 | Retrospective cohort | 26 | 37 | 25 | 74 | 15 | 64 | 25 |  |  |  |  | 5.6 |
| Saito et al (42) | 2015 | 145 | RCT | 145 |  |  | 136 | 9 | 129 | 16 |  |  |  |  | 24 |
| Salaun et al (43) | 2018 | 23 | Prospective cohort | 23 |  |  | 17 | 6 | 16 | 6 |  |  |  |  | 2 |
| Schleifer et al (44) | 2018 | 94 | RCT | 52 | 42 |  | 94 | 0 | 88 | 6 |  |  |  |  | 12 |
| Seo J et al (45) | 2020 | 429 | Retrospective cohort | 429 |  |  | 429 | 0 | 387 | 42 |  |  |  |  | 28.5 |
| Seo Y et al (46) | 2020 | 373 | Retrospective cohort |  |  |  | 373 | 0 | 324 | 49 |  |  |  |  | 11 |
| Shan et al (47) | 2018 | 16 | Prospective cohort |  |  |  |  |  |  |  | 1.1 | 1 | 0.8 | 1.1 | 24 |
| Stassen et al (48) | 2022 | 852 | Retrospective cohort |  |  | 852 | 668 | 184 | 658 | 194 |  |  |  |  | 6 |
| Upadhyay et al (122) | 2021 | 30 | Retrospective cohort | 8 | 18 | 22 |  |  |  |  |  |  |  |  | 4.7 |
| Vaidya et al LLPPM (49) | 2019 | 86 | Prospective cohort | 90 |  |  | 67 | 19 | 67 | 19 |  |  |  |  | 2 |
| Vaidya et al TVPPM (49) | 2019 | 89 | Prospective cohort | 90 |  |  | 60 | 29 | 49 | 40 |  |  |  |  | 6 |
| Van De Heyning et al (50) | 2019 | 328 | Prospective cohort | 174 | 107 | 47 | 274 | 16 | 260 | 30 |  |  |  |  | 12 |
| Vaturi et al (51) | 2010 | 23 | Prospective cohort |  |  |  | 14 | 9 | 5 | 18 |  |  |  |  | 48.6 |
| Wang et al (52) | 2023 | 8 | Prospective cohort | 8 | 0 | 0 | 1 | 7 | 6 | 2 |  |  |  |  | 3 |
| Webster et al (53) | 2007 | 123 | Retrospective |  |  |  | 123 | 0 | 92 | 31 |  |  |  |  | 27.2 |
| Wiechecka et al (54) | 2020 | 110 | Prospective cohort | 87 | 21 | 2 | 72 | 38 | 83 | 27 |  |  |  |  | 0.23 |
| Wu et al (55) | 2021 | 54 | Prospective cohort | 0 | 44 | 54 |  |  |  |  | 1.1 | 0.7 | 0.8 | 0.7 | 12 |
| Wu et al (55) | 2021 | 49 | Prospective cohort | 1 | 35 | 48 |  |  |  |  | 1 | 0.9 | 0.7 | 0.7 | 12 |
| Wu et al (55) | 2021 | 32 | Prospective cohort | 4 | 21 | 28 |  |  |  |  | 1.3 | 0.5 | 0.7 | 0.7 | 12 |
| Yu et al (56) | 2020 | 284 | Retrospective cohort | 284 |  |  | 249 | 35 | 138 | 146 |  |  |  |  | 55 |
| Zhang et al (57) | 2023 | 29 | Prospective cohort |  |  |  | 22 | 7 | 29 | 0 |  |  |  |  | 21 |

CIED: cardiac implantable electronic devices; CRT: Cardiac resynchronization therapy; DOF: duration of follow-up in months; ICD: implantable cardioverter defibrillator; PPM: permanent pacemaker; TR: Tricuspid regurgitation; (n) = number of significant TR events.

*Median and interquartile range

**Supplementary Table 4: Significant mitral regurgitation (MR) pre- and post- cardiac implantable electronic devices and duration of follow-up.**

| **Study** | **Year** | **Sample size** | **Study Design** | **PPM** | **ICD** | **CRT** | **Pre-CIED** | | **Post-CIED** | | **Pre-CIED** | | **Post-CIED** | | **DOF (months)** |
| --- | --- | --- | --- | --- | --- | --- | --- | --- | --- | --- | --- | --- | --- | --- | --- |
|  |  |  |  |  |  |  | ***No significant MR (n)*** | ***Significant MR (n)*** | ***No significant MR (n)*** | ***Significant MR (n)*** | ***Mean MR Grade*** | ***Standard Deviation MR Grade*** | ***Mean MR Grade*** | ***Standard deviation MR Grade*** |  |
| Alizadeh et al (4) | 2011 | 115 | Prospective cohort | 115 |  |  | 114 | 1 | 107 | 8 |  |  |  |  | 48.96 |
| Binda et al (65) | 2018 | 172 | Prospective registry |  |  | 172 | 132 | 40 | 155 | 17 |  |  |  |  | 9 |
| Boriani et al (67) | 2012 | 659 | Prospective registry |  |  | 659 | 427 | 232 | 422 | 136 |  |  |  |  | 6 |
| Brzezińska et al (70) | 2016 | 90 | Prospective cohort |  |  | 90 | 24 | 66 | 54 | 36 |  |  |  |  | 1.5 |
| Cabrera-Bueno et al (71) | 2010 | 76 | Prospective cohort |  |  | 76 | 44 | 32 | 48 | 28 |  |  |  |  | 6 |
| Chatterjee et al (72) | 2016 | 426 | Retrospective cohort |  |  | 426 | 201 | 225 | 226 | 200 |  |  |  |  | 6 |
| Chen et al (11) | 2022 | 51 | Prospective cohort | 16 | 35 | 51 |  |  |  |  | 1.68 | 0.95 | 1.54 | 0.92 | 12 |
| Cipriani et al (73) | 2016 | 1122 | Prospective cohort |  |  | 1122 | 508 | 614 | 681 | 235 |  |  |  |  | 12 |
| Chodor-Rozwadowska (13) | 2023 | 101 | Prospective cohort | 47 | 28 | 16 | 87 | 14 | 83 | 18 |  |  |  |  | 13 |
| Dabas et al (14) | 2021 | 42 | Retrospective cohort | 42 |  |  | 50 | 4 | 44 | 10 |  |  |  |  |  |
| Di Biase et al (76) | 2011 | 559 | Prospective cohort |  |  | 559 | 360 | 199 | 404 | 155 |  |  |  |  | 12 |
| Donnellan et al RVP (77) | 2019 | 53 | Retrospective cohort | 53 |  |  | 53 | 0 | 34 | 19 |  |  |  |  | 42 |
| Haeberlin et al (20) | 2022 | 69 | Retrospective cohort | 69 |  |  | 59 | 10 | 58 | 11 |  |  |  |  | 11.4 |
| Hai et al (21) | 2021 | 64 | Retrospective cohort | 64 |  |  | 51 | 6 | 49 | 15 |  |  |  |  | 14.7 |
| Hemayat et al RVA (23) | 2014 | 82 | Prospective cohort | 82 |  |  | 82 | 0.5 | 79 | 3 |  |  |  |  | 12 |
| Hemayat et al RVOT (23) | 2014 | 82 | Prospective cohort | 82 |  |  | 82 | 0.5 | 81 | 1 |  |  |  |  | 12 |
| Jin et al* (82) | 2022 | 46 | Prospective cohort | 46 |  |  |  |  |  |  | 1 | 1-2 | 1 | 1-2 | 12 |
| Jin et al* (82) | 2022 | 10 | Prospective cohort | 10 |  |  |  |  |  |  | 1 | 1-3 | 1.5 | 1-2 | 12 |
| Kelarijani et al (86) | 2008 | 60 | Prospective cohort |  |  | 60 | 24 | 36 | 55 | 5 |  |  |  |  | 6 |
| Mangiavacchi et al (95) | 2006 | 156 | Prospective cohort |  |  | 156 | 83 | 73 | 103 | 53 |  |  |  |  | 12 |
| Marincheva et al (35) | 2022 | 111 | Prospective cohort | 90 | 21 |  | 105 | 6 | 103 | 8 |  |  |  |  | 6 |
| Markman et al non-CRT (36) | 2020 | 478 | Retrospective cohort | 372 | 105 |  | 335 | 123 | 357 | 102 |  |  |  |  | 8.3 |
| Markman et al CRT (36) | 2020 | 105 | Retrospective cohort |  |  | 105 | 60 | 45 | 73 | 32 |  |  |  |  | 8.3 |
| Mihos et al (100) | 2017 | 71 | Retrospective cohort |  |  | 71 | 38 | 33 | 43 | 28 |  |  |  |  | 48 |
| Onishi et al (103) | 2013 | 240 | Prospective cohort |  |  | 240 | 126 | 114 | 150 | 90 |  |  |  |  | 6 |
| Rocha et al (109) | 2015 | 116 | Prospective cohort |  |  | 116 | 63 | 53 | 83 | 33 |  |  |  |  | 12 |
| Sadreddini et al (41) | 2014 | 89 | Retrospective cohort | 26 | 37 | 25 | 61 | 28 | 59 | 30 |  |  |  |  | 5.6 |
| Salaun et al (43) | 2018 | 23 | Prospective cohort | 23 |  |  | 18 | 5 | 17 | 5 |  |  |  |  | 2 |
| Sassone et al (110) | 2001 | 27 | Prospective cohort | 27 |  |  | 27 | 0 | 16 | 11 |  |  |  |  |  |
| Sénéchal et al (111) | 2010 | 57 | Prospective cohort |  |  | 57 | 26 | 31 | 46 | 11 |  |  |  |  | 6 |
| Shan et al (47) | 2018 | 16 | Prospective cohort |  |  |  |  |  |  |  | 1.7 | 0.8 | 1.3 | 0.8 | 24 |
| Solomon et al (114) | 2010 | 239 | RCT |  |  | 239 | 211 | 38 | 239 | 10 |  |  |  |  | 12 |
| Upadhyay et al (122) | 2021 | 30 | Retrospective cohort | 8 | 18 | 22 | 21 | 7 | 27 | 3 |  |  |  |  | 4.7 |
| Upadhyay et al (121) | 2015 | 439 | Retrospective cohort |  |  | 439 | 234 | 205 | 281 | 158 |  |  |  |  | 6 |
| van der Bijl et al (124) | 2019 | 1313 | Prospective cohort |  |  | 1313 | 675 | 518 | 818 | 375 |  |  |  |  | 6 |
| Verhaert et al (125) | 2012 | 266 | Prospective cohort |  |  | 266 | 226 | 40 | 194 | 30 |  |  |  |  | 6 |
| Wang et al (52) | 2023 | 8 | Prospective cohort | 8 |  |  | 7 | 1 | 8 | 0 |  |  |  |  | 3 |
| Wu et al (55) | 2021 | 32 | Prospective cohort | 4 | 21 | 28 |  |  |  |  | 1.7 | 1 | 0.7 | 0.8 | 12 |
| Wu et al (55) | 2021 | 49 | Prospective cohort | 1 | 35 | 48 |  |  |  |  | 1.8 | 0.9 | 0.9 | 0.8 | 12 |
| Wu et al (55) | 2021 | 54 | Prospective cohort | 0 | 44 | 54 |  |  |  |  | 2 | 0.8 | 1.1 | 0.9 | 12 |
| Zhang et al (57) | 2023 | 29 | Prospective cohort |  |  |  | 11 | 18 | 27 | 2 |  |  |  |  | 21 |

CIED: cardiac implantable electronic devices; CRT: Cardiac resynchronization therapy; DOF: duration of follow-up in months; ICD: implantable cardioverter defibrillator; PPM: permanent pacemaker; MR: mitral regurgitation; (n) = number of significant MR events.

*Median and interquartile range

**Supplementary Table 5:** **Mean grade of secondary mitral regurgitation (MR) pre- and post-cardiac resynchronization therapy.**

| **Author** | **Year** | **Sample size** | **Mean MR Grade pre-CRT (or CRT-off) ± standard deviation** | **Mean MR Grade post-CRT (or CRT-on) ± standard deviation** | **P value for change in MR grade** | **Post-implantation MR**  **Assessment time** |
| --- | --- | --- | --- | --- | --- | --- |
|  |  |  |  |  |  |  |
| Achilli et al (58) | 2006 | 133 | 2.2±1.0 | 1.8±0.9 | P = 0.003 | 6 months |
| Adelstein et al (60) | 2008 | 309 | 1.7±1.1 | 1.2 ± 1.1 | - | 18 months |
| Atta et al (62) | 2013 | 40 (QRS≥130 ms) | 2.3±0.6 | 1.5±1.0 | - | 6 months |
|  |  | 10 (QRS <130 ms) | 2.1±0.3 | 2.5±0.9 | - | 6 months |
| Chen et al BIV (11) | 2022 | 51 | 1.68±0.95 | 1.54±0.92 | - | 12 months |
| Delnoy et al (75) | 2007 | 167 (Sinus rhythm) | 1.7±1.2 | 1.0±1.1 | P<0.05 | 12 months |
|  |  | 96 (Atrial fibrillation) | 1.8±1.1 | 1.1±1.1 | P<0.05 | 12 months |
| Donnellan et al CRT (77) | 2019 | 25 | 3.5±1.4 | 2.6±0.9 | P=0.001 | 42 months |
| Iori et al (81) | 2014 | 42 | 2.8±0.6 | 2.3±0.9 | P=0.00998 | 26.2 months |
| Killu et al (87) | 2013 | 638 (≤80 years old) | 1.50 ± 0.78 | 1.27 ± 0.63 | P < 0.05 | 6.7 months |
|  | 2013 | 90 (>80 years old) | 1.60 ± 0.81 | 1.30 ± 0.68 | P < 0.05 |  |
| Kranig et al (88) | 2015 | 39 | 1.03 ± 0.72 | 0.56 ± 0.50 | p=0.003 | 12 months |
| Kuppahally et al (89) | 2009 | 35 (CRT-responders) | 1.18 ± 0.50 | 0.86 ± 0.56 | P= 0.01 | 23.7 months |
|  |  | 13 (CRT-nonresponders) | 1.38 ± 0.96 | 1.46 ± 0.87 | P> 0.05 |  |
| Mihos et al (100) | 2017 | 71 | 1.9 ± 1.0 | 1.5 ± 1.0 | P = .001 | 48 months |
| Naqvi et al (102) | 2008 | 18 (Improved MR) | 2.78 ± 0.65 | 0.86 ±0.54 | P< 0.0001 | 1.7 months |
|  | 2008 | 17 (Unimproved MR) | 3.65 ± 0.61 | 2.94 ± 0.83 | P=0.002 |  |
| Praus et al (106) | 2012 | 58 | 2.6 ± 0.9 | 2.2 ± 0.9 | P< 0.001 | 3 months |
| Reuter et al (108) | 2000 | 47 | 1.8 ± 1.0 | 1.3 ± 0.7 | P < 0.01 | 3 months |
| Rao et al (107) | 2007 | 101 (LV pacing) | 1.22±0.69 | 1.19±0.72 | P= 0.66 | 6 months |
|  |  | 104 (Sequential BIV) | 1.26±0.62 | 0.97± 0.60 | P<0.001 |  |
|  |  | 101 (Simultaneous BIV) | 1.07±0.70 | 0.83 ±0.58 | P<0.001 |  |
| Stockburger et al (118) | 2008 | 21 | 1.2 ± 0.6 | 0.8 ± 0.7 | P = 0.02 | 20.6 months |
| Wu et al BIV (55) | 2021 | 54 | 2.0±0.8 | 1.1±0.9 | - | 12 months |
| Ypenburg et al (128) | 2008 | 29 (Early MR responders) | 2.8+0.6 | 1.6+0.8 | P<0.001 | 6 months |
|  |  | 14 (Late MR responders) | 2.8+0.7 | 2.8+0.7 | P<0.001 |  |
|  |  | 25 (Non-MR responders) | 2.8+0.7 | 2.8+0.7 | P=0.4 |  |
| Zanon et al (130) | 2004 | 45 | 2.06 ± 0.83 | 1.42 ± 0.61 | P < 0.001 | 0.25 months |

For Rao et al (107), the LV only pacing group was not included in the meta-analysis.

**Supplementary Table 6: Mean effective regurgitant orifice area (EROA) in mm^2^ of secondary mitral regurgitation (MR) pre- and post-cardiac resynchronization therapy.**

| **Author** | **Year** | **Sample size** | **Mean MR EROA pre-CRT (or CRT-off) ± standard deviation** | **Mean MR EROA post-CRT (or CRT-on) ± standard deviation** | **P value for change in MR EROA** | **Post-implantation MR**  **Assessment time** |
| --- | --- | --- | --- | --- | --- | --- |
|  |  |  |  |  |  |  |
| Brandt et al (68) | 2006 | 20 | @EROA 9.1 (5.7–13.3) mm2 | EROA 4.8 (0.0–7.8) mm2 | P= 0.0001 | 14.3 months |
| Breithardt et al (69) | 2003 | 24 | EROA 25 +/- 19 mm2 | EROA 13 +/- 8 mm2 | P<0.001 | Acutely |
| Cleland et al (CARE-HF trial) (74) | 2008 | 813 (409 CRT) | @EROA 21.3 (11.6-32.9) mm2 | EROA 14.1 (7.6-24.8) mm2 | P= 0.0002 | 3 months |
| Ennezat et al (78) | 2006 | 21 | EROA 11 ±2 mm2 | EROA 8 ±2 mm2 | p = 0.02 | Acutely |
| Karvounis et al (85) | 2006 | 22 | EROA 35 ± 17 mm2 | EROA 18 ±11 mm2 | *P* < .001 | Acutely |
| Lancellotti et al (90) | 2004 | 27 | EROA 22 ±10 mm2 | EROA 13 ±7 mm2 | P= 0.00001 | 1.5 months |
| Madaric et al (94) | 2007 | 28 | EROA 25+12 mm2 | EROA 13+7 mm2 | P <0.001 | 3 months |
| Marechaux et al (96) | 2009 | 26 | EROA 9 ± 7 mm2 | EROA 8 ± 7 mm2 | P = .05 | Acute |
| Martens et al (97) | 2018 | 31 | EROA  -Rest 32±10 mm2  -Exercise 48±22 mm2 | EROA  -Rest 14±2 mm2  -Exercise 17±1 mm2 | p<0.001  p <0.001 | 6 months |
| Porciani et al (105) | 2006 | 15 (CRT responders) | EROA 20.8 ± 11.9 mm2 | EROA 14.4 ± 7.6 mm2 | P>0.05 (NS) | 6 months |
|  | 2006 | 15 (CRT non-responders) | EROA 34.9 ± 21.6 mm2 | EROA 25.3 ±7.7 mm2 | P>0.05(NS) |  |
| Sénéchal et al (111) | 2010 | 57 | EROA 20±12 mm2 | EROA 12±11 mm2 | P=0.001 | 6 months |
| Sitgesat al (112) | 2009 | 57 | EROA 32 ±16 mm2 | EROA 19 ±17mm2 | P <0.01 | 12 months |
| Stolfo et al (119) | 2015 | 44 | EROA 22 ± 6 mm2 | EROA 18 ± 8 mm2 | P = 0.006 | 7 months |
| Tournoux et al (120) | 2007 | 53 | EROA 17+12 mm2 | EROA 17+20 mm2 | P = 0.92 | 9 months |
| van Bommel et al (123) | 2011 | 98 | EROA 51±16 mm2 | EROA 43±18 mm2 | P=0.001 | 6 months |
| Vinereanu et al (126) | 2007 | 22 | EROA 15.3± 12 mm2 | EROA 10.5 ± 9 mm2 | P < .05 | - |
| Witte et al (127) | 2008 | 11 | EROA 17±15 mm2 | EROA 13±3 mm2 | P<0.05 | 1.5 months |

^@^Median and interquartile range. Two studies (Brandt et al (68) & Cleland et al (74)) from Supplementary Table 3 were excluded as they reported median and interquartile range instead of mean and standard deviation.

**Supplementary Table 7: Mean regurgitant volume (RV) and regurgitant fraction (RF) of secondary mitral regurgitation (MR) pre- and post-cardiac resynchronization therapy.**

| Author | Year | Sample size | **Mean MR regurgitant volume or fraction pre-CRT (or CRT-off) ± standard deviation** | **Mean MR regurgitant volume or fraction post-CRT (or CRT-on) ± standard deviation** | P value for change in MR | Post-implantation MR  Assessment time |
| --- | --- | --- | --- | --- | --- | --- |
|  |  |  |  |  |  |  |
| Brandt et al (68) | 2006 | 20 | @RV 16.0 (10.7– 20.8) ml | RV 7.8 (0.0–11.5) ml | P=0.0001 | 14.3 months |
|  |  |  | @RF 27.7 (14.6– 34.0) % | RF 13.8 (0.0–19.2) % | P=0.0002 |  |
| Breithardt et al (69) | 2003 | 24 | RV 32 ± 19 ml | RV 19 ± 9 ml | P<0.001 | Acutely |
| Ennezat et al (78) | 2006 | 21 | RV 18 ±3 ml | RV 13 ±3ml | p = 0.03 | Acutely |
| Fukuda et al (79) | 2005 | 19 | RV 61.6 ±19.8 ml | RV 41.2 ± 14.1 ml | P= 0.002 | Acutely |
|  |  |  | RF 51.6 ±8.3% | RF 39.8 ± 13.1% | p =0.002 |  |
| Kanzaki et al (83) | 2004 | 26 | RV 40 ±20 ml | RV 24 ± 17 ml | P< 0.001 | 1-day post-CRT |
|  |  |  | RF 40 ±12% | RF 25 ± 14% | P<0.001 |  |
| Karvounis et al (85) | 2006 | 22 | RV 35±15 ml | RV 26 ± 12 ml | P=0.005 | Acutely |
| Liang et al (92) | 2010 | 83 | RV 38+20 ml | RV 33+21 mL | P < 0.05 | 3months |
| Madaric et al (94) | 2007 | 28 | RV 36+19 ml | RV 25+11 ml | P<0.001 | 3 months |
|  |  |  | RF 53+12 % | RF 39+20 % | P=0.031 |  |
| Marechaux et al (96) | 2009 | 26 | RV 15 ± 11 ml | RV 12 ± 10 ml | P = .015, | Acute |
| Sénéchal et al (111) | 2010 | 57 | RV 33 ±27 ml | RV 23 ±20 ml | P =0.006 | 6 months |
| Sitges et al (112) | 2009 | 57 | RV 46 ±21 ml | RV 28 ± 22 ml | P <0.01 | 12 months |
| Solis et al (113) | 2009 | 34 | RV 35±17 ml | RV 23±14 ml | P < 0.001 | 6 months |
| Witte et al (127) | 2008 | 11 | RV 24±27 ml | RV 22.0±5.4 ml | P<0.05 | 1.5 months |

^@^Median and interquartile range. One study (Brandt et al (68)) from Supplementary Table 3 was excluded as they reported median and interquartile range instead of mean and standard deviation.

**Supplementary Table 8: Mean mitral regurgitant jet area (MRJA) in cm^2^ of secondary mitral regurgitation (MR) pre- and post-cardiac resynchronization therapy.**

| **Author** | **Year** | **Sample size** | **Mean MR mitral regurgitant jet area (MRJA) pre-CRT (or CRT-off) ± standard deviation** | **Mean MR mitral regurgitant jet area (MRJA) post-CRT (or CRT-on) ± standard deviation** | **P value for change in MR MRJA** | **Post-implantation MR**  **Assessment time** |
| --- | --- | --- | --- | --- | --- | --- |
|  |  |  |  |  |  |  |
| Achilli et al (59) | 2008 | 106 | MRJA 7.4±5.1 cm2 | MRJA 4.5±3.8 cm2 | p<0.001 | 12 months |
| António et al (61) | 2010 | 51 (Young) | MRJA 8.1 cm2 | MRJA 6.2 cm2 | P <0.05 | 6 months |
|  | 2010 | 36 (Elderly) | MRJA 7.6 cm2 | MRJA 5.4 cm2 | P<0.05 |  |
| Brandt et al (68) | 2006 | 20 | @MRJA 5.9 (2.2–7.5) cm2 | MRJA 4.1 (0.8–6.3) cm2 | P=0.002 | 14.3 months |
| Gras et al (80) | 2002 | 46 | MRJA 7.66±5.5 cm2 | MRJA 6.69±5.9 cm2 | P= 0.197 | 12 months |
| Linde et al (93) | 2002 | 67 (Sinus rhythm) | MRJA 7.4 ±6.8 cm2 | MRJA 4.3±4.0 cm2 | - | 12 months |
|  |  | 64 (Atrial fibrillation) | MRJA 10.2 ±13.7cm2 | MRJA 5.4 ±3.9cm2 | - | 12 months |
| Menardi et al (99) | 2008 | 100 | MRJA 13.3± 4.2 cm2 | MRJA 6.1 ± 4.1 cm2 | P= 0.001 | 12 months |
| Porciani et al (105) | 2006 | 15 (CRT responders) | MRJA 6.9±3.8 cm2 | MRJA 3.7±2.4 cm2 | P<0.01 | 6 months |
|  | 2006 | 15 (CRT non-responders) | MRJA 8.8±4.4 cm2 | MRJA 7.2±3.3 cm2 | P>0.05 (NS) |  |
| Stellbrink et al (115) | 2001 | 25 | MRJA 3.8±3.6cm2 | MRJA 3.3±2.1 cm2 | NS | 6 months |
| St John Sutton et al (MIRACLE Study) (116) | 2006 | 176 | MRJA 7.41±6.1 cm2 | MRJA 4.4±4.2 cm2 | P<0.001 | 12 months |
| St John Sutton et al (REVERSE Study) (117) | 2009 | 419 | MRJA 15.1±11.5 cm2 | MRJA 12.5±10.8 cm2 | P > 0.05 (NS) | 12 months |
| Stolfo D et al (119) | 2015 | 44 | MRJA 9.2 ± 4.1 cm2 | MRJA 7.4 ± 5.1 cm2 | - | 7 months |
| Witte et al (127) | 2008 | 11 | MRJA 5.0±4.0 cm2 | MRJA 3.7±1.1 cm2 | P<0.05 | 1.5 months |
| Ypenburg et al (128) | 2008 | 29 (Early MR responders) | MRJA 7.1+3.2 cm2 | MRJA 3.0+1.3 cm2 | P<0.001 | 6months |
|  |  | 14 (Late MR responders) | MRJA 7.9+4.0 cm2 | MRJA 3.7+2.1 cm2 | P<0.001 |  |
|  |  | 25 (Non MR responders) | MRJA 7.0+4.7 cm2 | MRJA 8.5+4.9 cm2 | P=0.3 |  |
| Zanon et al (130) | 2004 | 45 | MRJA 6.81 ± 4.83 cm2 | MRJA 4.22 ± 3.62 cm2 | P < 0.001 | 0.25 months |
| Karaca et al (84) | 2018 | 50 (MR responders) | *MVTA 4.15 ± 1.05 cm2 | MVTA 3.67 ± 1.01 cm2 | P < 0.001 | 6 months |
|  |  | 45 (MR non-responders) | *MVTA 3.68 ± 1.04 cm2 | MVTA 3.98 ± 0.97 cm2 | P= 0.014 |  |
| Martens et al (97) | 2018 | 31 | *MVTA 3.4±0.4 cm2 | MVTA 1.9±0.7 cm2 | p<0.001 | 6 months |
| Sitges et al (112) | 2009 | 57 | *MVTA 3.36±0.98 cm2 | MVTA 2.78 ±0.75 cm2 | P <0.01 | 12 months |
| Solis et al (113) | 2009 | 34 | ∑MVAA 11.6±3.5 cm2 | MVAA 10.5±3.1 cm2 | P < 0.001 | 6 months |
| Solomon et al (114) | 2010 | 239 out of 749 | &MRJW 0.22±0.12 cm | MRJW 0.18±0.08 cm | P=0.003 | 12 months |

MRJA Mitral regurgitant jet area

^@^Median and interquartile range

*MTA Mitral tenting area

^∑^MVAA Mitral valve annular area

^&^MRJW Mitral regurgitation jet width

NS Not significant

**Supplementary Table 9: Mean proportion of mitral regurgitant jet area to left atrial area (MRJA/LAA %) of secondary mitral regurgitation (MR) pre- and post-cardiac resynchronization therapy.**

| **Author** | **Year** | **Sample size** | **Mean MR MRJA/LAA % pre-CRT (or CRT-off) ± standard deviation** | **Mean MR MRJA/LAA % post-CRT (or CRT-on) ± standard deviation** | **P value for change in MR** | **Post-implantation MR**  **Assessment time** |
| --- | --- | --- | --- | --- | --- | --- |
|  |  |  |  |  |  |  |
| Bakker et al (63) | 2000 | 12 | MRJA/LAA % 41 ± 16 | MRJA/LAA % 29 ± 12 | P=0.03 | 12 months |
| Bordachar et al (66) | 2004 | 33 | MRJA/LAA % 37.9 ± 14 | MRJA/LAA% 24.7 ± 11 | P *<* 0.01 | 2 days post-implantation |
| Brandt et al (68) | 2006 | 20 | @MRJA/LAA % 20.3 (13.1–26.1) | MRJA/LAA% 13.8 (4.1–23.1) | P=0.0001 | 14.3 months |
| Lau et al (91) | 2000 | 11 | MRJA/LAA% 46 ±25 | MRJA/LAA% 26 ± 20 | P < 0.05 | 3 months |
| Matsumoto et al (98) | 2011 | 31 (CRT responders) | MRJA/LAA% 38 ±13 | MRJA/LAA% 12 ± 10 | p <0.001 | 6 months |
|  | 2011 | 13 (CRT non-responders) | MRJA/LAA% 38 ±9 | MRJA/LAA% 36 ±9 | p >0.05 (NS) |  |
| Naqvi et al (102) | 2008 | 18 (Improved MR) | MRJA/LAA% 33.7 ± 5.2 | MRJA/LAA% 12.5 ± 9.2 | P <0.0001 | 1.7 months |
|  | 2008 | 17 (Unimproved MR) | MRJA/LAA% 47.4 ±12.5 | MRJA/LAA% 36.3 ±11.3 | P= 0.00006 |  |
| Porciani et al (105) | 2006 | 15 (CRT responders) | MRJA/LAA% 30±19 | MRJA/LAA% 15±6 | P<0.05 | 6 months |
|  | 2006 | 15 (CRT non-responders) | MJA/LAA% 32±14 | MJA/LAA% 27±9 | P>0.05 (NS) |  |
| Vinereanu et al (126) | 2007 | 22 | MRJA/LAA% 32 ±13 | MRJA/LAA% 26 ± 14 | P < .01 | - |
| Ypenburg et al (128) | 2008 | 29 (Early MR responders) | MRJA/LAA% 34+13 | MRJA/LAA% 17+8 | P<0.001 | 6months |
|  |  | 14 (Late MR responders) | MRJA/LAA% 34+15 | MRJA/LAA% 20+11 | P<0.001 |  |
|  |  | 25 (Non-MR responders) | MRJA/LAA% 35+15 | MRJA/LAA% 35+15 | P=0.7 |  |
| Yu et al (129) | 2006 | 51 | MRJA/LAA% 34 ±19 | MRJA/LAA% 21 ±18 | P<0.001 | 3 months |
| Zanon et al (130) | 2004 | 45 | MRJA/LAA% 22.8 ± 13.11 | MRJA/LAA% 14.53 ± 11.77 | P < 0.001 | 0.25 months |

**Supplementary Table 10: Mean vena contracta (VC) of secondary mitral regurgitation (MR) pre- and post-cardiac resynchronization therapy.**

| **Author** | **Year** | **Sample size** | **Mean MR vena contracta pre-CRT (or CRT-off) ± standard deviation** | **Mean MR vena contracta post-CRT (or CRT-on) ± standard deviation** | **P value for change in MR** | **Post-implantation MR**  **Assessment time** |
| --- | --- | --- | --- | --- | --- | --- |
|  |  |  |  |  |  |  |
| Matsumoto et al (98) | 2011 | 31 (CRT responders) | VC 0.67 ± 0.19 cm | VC 0.21± 0.19 cm | p <0.001 | 6 months |
|  | 2011 | 13 (CRT non-responders) | VC 0.68 ± 0.15 cm | VC 0.64 ±0.13 cm | p >0.05 (NS) |  |
| Menardi et al (99) | 2008 | 100 | VC 0.55± 0.1 cm | VC 0.32± 0.1 cm | P= 0.001 | 12 months |
| Mihos et al (100) | 2017 | 71 | VC 0.37 ± 0.18 cm | VC 0.29 ± 0.20 cm | *P* = .001 | 48 months |
| Stolfo et al (119) | 2015 | 44 | VC 0.67 ± 0.21 cm | VC 0.56 ± 0.31 cm | P = 0.008 | 7 months |
| van Bommel et al (123) | 2011 | 98 | VC 0.74±0.15 cm | VC 0.59±0.21 cm | P<0.001 | 6 months |
| Verhaert et al (125) | 2012 | 266 | VC 0.35±0.24 cm | VC 0.27 ±0.24 cm | P<0.0001 | 6 months |
| Vinereanu et al (126) | 2007 | 22 | VC 0.52 ± 0.17 cm | VC 0.40 ±0.17 cm | P < .05 | - |
| Ypenburg et al (128) | 2008 | 29 (Early MR responders) | VC 0.46+0.16 cm | VC 0.31+0.10 cm | P<0.001 | 6months |
|  |  | 14 (Late MR responders) | VC 0.46+0.22 cm | VC 0.27+0.14 cm | P<0.001 |  |
|  |  | 25 (Non-MR responders) | VC 0.42+0.20 cm | VC 0.44+0.18 cm | P=0.3 |  |

**Supplementary Table 11: All-Mortality risk associated with significant TR post- cardiac implantable electronic devices.**

| **Study** | **Year** | **Sample size** | **Study Design** | **PPM** | **ICD** | **CRT** | **No TR Pre-CIED** | **TR Pre-CIED** | **No TR Post-CIED** | **TR Post-CIED** | **DOF** | **Hazard ratio death** | **Lower CI death** | **Upper CI death** | **P-value death** | **DOF till death** |
| --- | --- | --- | --- | --- | --- | --- | --- | --- | --- | --- | --- | --- | --- | --- | --- | --- |
| Abu Sham’a et al (1) | 2013 | 193 | Prospective cohort | - | - | 193 | 158 | 35 | 133 | 60 | 12 | 6.7 | 1.8 | 24.5 | 0.004 |  |
| Al-bawardy et al (3) | 2015 | 1596 | Retrospective cohort | 310 | 985 | 334 | 736 | 860 | 511 | 1085 | 48 | 1.72 | 1.5 | 1.99 | 0.0001 | 48 |
| Delling et al (15) | 2016 | 634 | Retrospective cohort | 634 | - | - | 621 | 13 | 533 | 101 | 15.6 | 1.4 | 1.04 | 2.11 | - |  |
| Grupper et al (18) | 2015 | 689 | Retrospective cohort | - | - | 689 | 447 | 242 | 585 | 104 | 15 | 1.36 | 0.88 | 2.1 | 0.17 | 36.9 |
| Hoke et al (24) | 2014 | 239 | Retrospective cohort | 48 | 191 |  | 212 | 27 | 128 | 111 | 18 | 1.749 | 1.008 | 3.055 | 0.047 | 70 |
| Papageorgiou et al (38) | 2020 | 304 | Retrospective cohort | 95 | 203 | 6 | 304 | 0.5 | 238 | 66 | 138.2 | 3.14 | 1.29 | 7.63 | 0.01 | 138 |
| Riesenhuber et al (39) | 2021 | 990 | Retrospective cohort | 990 | - | - | 727 | 263 | 551 | 439 | 5 | 1.38 | 1.04 | 1.84 | 0.028 | 67.2 |
| Stassen et al (48) | 2022 | 852 | Retrospective cohort | - | - | 852 | 668 | 184 | 658 | 194 | 6 | 1.745 | 1.287 | 2.366 | 0.001 | 92 |

CIED: cardiac implantable electronic devices; CRT: Cardiac resynchronization therapy; DOF: duration of follow-up in months; ICD: implantable cardioverter defibrillator; PPM: permanent pacemaker; TR: Tricuspid regurgitation

**Supplementary Table 12: Risk of all-cause mortality associated with persistent significant mitral regurgitation (MR) post-cardiac resynchronization therapy.**

| **Study** | **Year** | **Sample size** | **Significant MR pre-CRT (N)** | **Median duration of FU for MR assessment post-CRT** | **Significant MR post-CRT (N)** | **Outcome events during follow-up (N)** | **Median duration of follow-up for outcomes** | **HR (95% CI) of all-cause mortality in unimproved compared to improved SMR group** |
| --- | --- | --- | --- | --- | --- | --- | --- | --- |
| Binda et al (65) | 2018 | 172 | 40 | 9 months | 17 | 49 deaths,  36 HFH | 48 months | HR 3.77 (95% CI 1.30—10.92), p = 0.014 |
| BORIANI et al (67) | 2012 | 659 | 232 | 6 months | 136 | 66 deaths | 14-16 months | HR 0.7 (95% CI 0.4–1.5), p=0.399 |
| Cabrera-Bueno et al (71) | 2010 | 76 | 32 | 6 months | 28 | 22 events (10 deaths, 10 HFH, 2  transplants) | 19± 4 months | *HR 2.54 (95% CI 1.23-5.00), p = 0.011 (46.4% rate in unimproved MR vs. 18.7% in improved MR) |
| Cipriani et al (73) | 2016 | 1122 | 614 | 12 months | 235 | 205 deaths | 38.6 months | HR 1.58 (95% CI 1.09–2.28), p=0.015 |
| Cleland et al (74) | 2008 | 409 | - | 3 months | - | 101 deaths | 37.6 months | ^#^HR 2.673 (95% CI 1.881-3.799), p<0.0001 |
| Onishi et al (103) | 2013 | 240 | 114 | 6 months | 90 | 66 events (46 deaths, 10 transplants, 9 LVAD) | 48 months | ^#^HR 3.58 (95% CI, 2.18–5.87), p<0.0001 |
| Rocha et al (109) | 2015 | 116 | 53 | 12 months | 33 | 29 deaths | 34.1 months | HR 7.12 (95% CI 2.26-22.36), p=0.001 |
| Stolfo et al (119) | 2015 | 44 | 44 | 7 months | 23 | 8 deaths | 59 months | ^&^HR 2.6 (95% CI 1.15-5.89), p= 0.022 (90% survival for improved MR vs 74% for unimproved MR) |
| Upadhyay et al (121) | 2015 | 439 | 205 | 6 months | 158 | - | 36 months | ^&^HR 1.25 (95% CI 1.05-1.41), p=0.016 |
| van Bommel et al (123) | 2011 | 98 | 98 | 6 months | 56 | 34 deaths | 32 months | HR 2.86 (1.06-7.68), p=0.043 |
| van der Bijl et al (124) | 2019 | 1313 | 518 | 6 months | 375 | 297 deaths | 51 months | HR 1.77 (95% CI 1.41-2.22), p<0.001 |
| Verhaert et al (125) | 2012 | 266 | 40 | 6 months | 30 | 98 events (78 deaths, 14 transplants, 3 LVADs) | 42 months | ^#^HR 1.56 (95% CI 1.21-2.01), P =0.03 |

*HR Death or heart transplant or readmission for HF

^#^HR for death, heart transplantation or left ventricular assist device

^&^HR mortality or heart transplant

CI = Confidence interval; FU = follow-up; HFH = heart failure hospitalization; HR = Hazard ratio; LVAD = left ventricular assist device; MR = mitral regurgitation; N=number

**Supplementary Figure 2**: ***Proportion (prevalence) of significant TR pre-CIED implantation***

******

**Supplementary Figure 3**: ***Proportion (prevalence) of significant TR post-CIED implantation***

******

**Supplementary Figure 4**: ***Proportion (prevalence) of significant MR pre-CRT implantation***

**Supplementary Figure 5**: ***Proportion (prevalence) of significant MR post-CRT implantation***

**Supplementary Figure 6: Standardized mean difference (SMD) and 95% confidence interval (95% CI) in secondary mitral regurgitation (MR) grade *(10A)* and effective regurgitant orifice area (EROA*) (10B)* pre- and post-cardiac resynchronization therapy (CRT).** Values of MR grade and EROA are mean and standard deviation (SD); N = number of patients.

**Standardized mean difference (SMD) of MR grade post-CRT**

**Standardized mean difference (SMD) of MR EROA post-CRT**

**A**

**B**

**Supplementary Figure 7**. **Standardized mean difference (SMD) in mitral regurgitation (MR) regurgitant volume *(Figure 6A)*, and regurgitant fraction *(Figure 6B)* fraction pre- and post-cardiac resynchronization therapy.**

**Standardized mean difference (SMD) of MR regurgitant volume post-CRT**

**Standardized mean difference (SMD) of MR regurgitant fraction post-CRT**

**B**

**A**

**Supplementary Figure 8**: **Standardized mean difference (SMD) and 95% confidence interval (95% CI) in secondary mitral regurgitation (MR) mitral regurgitant jet area (MRJA) & in proportion of mitral regurgitant jet area to left atrial area (MRJA/LAA %) pre- and post-cardiac resynchronization therapy (CRT).**

**Standardized mean difference (SMD) of MRJA regurgitant volume post-CRT**

**Standardized mean difference (SMD) of MRJA/LAA % regurgitant volume post-CRT**

**A**

**B**

**Supplementary Figure 9.** **Standardized mean difference (SMD) in mitral regurgitation (MR) vena contracta pre- and post-cardiac resynchronization therapy.**

**Supplementary Figure 10: Risk of significant Tricuspid regurgitation (TR) post-implantable cardioverter defibrillator (ICD) devices compared to permanent pacemakers (PPM).**

**Risk of tricuspid regurgitation post-ICD compared to post-PPM**

**A**

**TR Post-All CIED**

**TR Post-RVP**

**B**

**TR Post-CSP**

**D**

**TR Post-LP**

**E**

**F**

**TR Post-CIED & mortality**

**TR Post-CRT**

**C**

**Supplementary Figure 11: Funnel plots of studies included in meta-analysis of tricuspid regurgitation (TR) post-cardiac implantable electronic devices (CIED).** Panels A to E depict funnel plots from odds ratio of significant TR post-CIED compared to pre-CIED. ***A:*** TR post-all CIED (Egger test p-value <0.001); ***B:*** TR post pure transvenous right ventricular pacing (RVP) via CIED with trans-tricuspid right ventricular leads (p <0.001); ***C:*** TR post-cardiac resynchronization therapy (CRT) (p=0.8323); ***D:*** TR post-conduction system pacing (CSP) (p=0.0042); ***E:*** TR post-Leadless pacing (LP) (p=0.9166). Panel ***F*** shows the funnel plot from hazard ratio of all-cause mortality in patients with significant TR post-CIED (p=0.0884). For any asymmetry in the funnel plot or significant Egger’s test p-value, trim and fill analysis showed no significant change in results suggesting less likelihood of publication bias.

**F**

**E**

**D**

**MR Post-CRT**

**MR Post-CRT & All-cause mortality**

**MR Post-LP**

**MR Post-CSP**

**C**

**A**

**MR Post-All CIED**

**MR Post- RVP**

**B**

**Supplementary Figure 12: Funnel plots of studies included in meta-analysis of mitral regurgitation (MR) post-cardiac implantable electronic devices (CIED).** Panels A to E depict funnel plots from odds ratio of significant MR post-CIED compared to pre-CIED. ***A:*** MR post-all CIED (Egger test p-value <0.001); ***B:*** MR post- right ventricular pacing (RVP) via CIED with trans-tricuspid right ventricular leads (Egger test p-value=0.0005); ***C:*** MR post-cardiac resynchronization therapy (CRT) (p=0.0082); ***D:*** MR post-conduction system pacing (CSP) (p=0.7898); ***E:*** MR post-Leadless pacing (LP) (p=0.9468). Panel ***F*** shows funnel plot from hazard ratio of all-cause mortality in patients with significant MR post-CRT (p=0.0991). For any asymmetry in the funnel plot or significant Egger’s test p-value, trim and fill analysis showed no significant change in results suggesting less likelihood of publication bias.

**TR Post-All CIED**

**MR Post-CRT & all-cause mortality**

**TR Post-CIED & all-cause mortality**

**TR Post-RVP**

**A**

**B**

**C**

**F**

**MR Post- RVP**

**D**

**MR Post-All CIED**

**E**

**Supplementary Figure 13: Contour-enhanced funnel plots of tricuspid regurgitation (TR) *(panels A to C),* and mitral regurgitation (MR) *(panels D to F)* post-cardiac implantable electronic devices (CIED).** CRT: Cardiac resynchronization therapy; RVP: Right ventricular pacing via CIED with trans-tricuspid right ventricular leads. For any discordant distribution of studies in contour-enhanced funnel plot, trim and fill analysis showed no significant change in results suggesting less likelihood of publication bias.

**REFERENCES**

1. Abu Sham'a R, Buber J, Grupper A, Nof E, Kuperstein R, Luria D, et al. Effects of tricuspid valve regurgitation on clinical and echocardiographic outcome in patients with cardiac resynchronization therapy. Europace. 2013;15(2):266-72.

2. Addetia K, Maffessanti F, Mediratta A, Yamat M, Weinert L, Moss JD, et al. Impact of implantable transvenous device lead location on severity of tricuspid regurgitation. J Am Soc Echocardiogr. 2014;27(11):1164-75.

3. Al-Bawardy R, Krishnaswamy A, Rajeswaran J, Bhargava M, Wazni O, Wilkoff B, et al. Tricuspid regurgitation and implantable devices. Pacing Clin Electrophysiol. 2015;38(2):259-66.

4. Alizadeh A, Sanati HR, Haji-Karimi M, Yazdi AH, Rad MA, Haghjoo M, et al. Induction and aggravation of atrioventricular valve regurgitation in the course of chronic right ventricular apical pacing. EP Europace. 2011;13(11):1587-90.

5. Anvardeen K, Rao R, Hazra S, Hay K, Dai H, Stoyanov N, et al. Prevalence and Significance of Tricuspid Regurgitation Post-Endocardial Lead Placement. JACC Cardiovasc Imaging. 2019;12(3):562-4.

6. Arabi P, Özer N, Ateş AH, Yorgun H, Oto A, Aytemir K. Effects of pacemaker and implantable cardioverter defibrillator electrodes on tricuspid regurgitation and right sided heart functions. Cardiol J. 2015;22(6):637-44.

7. Arps K, Li B, Allen JC, Jr., Alenezi F, Frazier-Mills C, Al-Khatib SM, et al. Association of leadless pacing with ventricular and valvular function. J Cardiovasc Electrophysiol. 2023;34(11):2233-42.

8. Baquero GA, Yadav P, Skibba JB, Banchs JE, Linton-Frazier LN, Lengerich EJ, et al. Clinical significance of increased tricuspid valve incompetence following implantation of ventricular leads. J Interv Card Electrophysiol. 2013;38(3):197-202.

9. Beurskens NEG, Tjong FVY, de Bruin-Bon RHA, Dasselaar KJ, Kuijt WJ, Wilde AAM, et al. Impact of Leadless Pacemaker Therapy on Cardiac and Atrioventricular Valve Function Through 12 Months of Follow-Up. Circ Arrhythm Electrophysiol. 2019;12(5):e007124.

10. Breeman KTN, Peijster AJL, De Bruin-Bon H, Pepplinkhuizen S, Van der Stuijt W, De Veld JA, et al. Worsening tricuspid regurgitation after ICD implantation is rather due to transvenous lead than natural progression. Int J Cardiol. 2023;376:76-80.

11. Chen X, Ye Y, Wang Z, Jin Q, Qiu Z, Wang J, et al. Cardiac resynchronization therapy via left bundle branch pacing vs. optimized biventricular pacing with adaptive algorithm in heart failure with left bundle branch block: a prospective, multi-centre, observational study. Europace. 2022;24(5):807-16.

12. Cho MS, Kim J, Lee JB, Nam GB, Choi KJ, Kim YH. Incidence and predictors of moderate to severe tricuspid regurgitation after dual-chamber pacemaker implantation. Pacing Clin Electrophysiol. 2019;42(1):85-92.

13. Chodór-Rozwadowska K, Sawicka M, Morawski S, Kalarus Z, Kukulski T. Tricuspid Regurgitation (TR) after Implantation of a Cardiac Implantable Electronic Device (CIED)-One-Year Observation of Patients with or without Left Ventricular Dysfunction. J Cardiovasc Dev Dis. 2023;10(8).

14. Dabas N, Penalver J, Colombo R, Mendoza I. OUTCOMES OF LEADLESS PACING ON TRICUSPID VALVE AND RIGHT VENTRICULAR FUNCTION. Journal of the American College of Cardiology. 2021;77(18_Supplement_1):247-.

15. Delling FN, Hassan ZK, Piatkowski G, Tsao CW, Rajabali A, Markson LJ, et al. Tricuspid Regurgitation and Mortality in Patients With Transvenous Permanent Pacemaker Leads. Am J Cardiol. 2016;117(6):988-92.

16. Fanari Z, Hammami S, Hammami MB, Hammami S, Shuraih M. The effects of right ventricular apical pacing with transvenous pacemaker and implantable cardioverter defibrillator on mitral and tricuspid regurgitation. J Electrocardiol. 2015;48(5):791-7.

17. Grieco D, Bressi E, Curila K, Padala SK, Sedlacek K, Kron J, et al. Impact of His bundle pacing on right ventricular performance in patients undergoing permanent pacemaker implantation. Pacing Clin Electrophysiol. 2021;44(6):986-94.

18. Grupper A, Killu AM, Friedman PA, Abu Sham'a R, Buber J, Kuperstein R, et al. Effects of tricuspid valve regurgitation on outcome in patients with cardiac resynchronization therapy. Am J Cardiol. 2015;115(6):783-9.

19. Hu Q, Lu W, Chen K, Dai Y, Lin J, Xu N, et al. Long-term follow-up results of patients with left bundle branch pacing and exploration for potential factors affecting cardiac function. Front Physiol. 2022;13:996640.

20. Haeberlin A, Bartkowiak J, Brugger N, Tanner H, Wan E, Baldinger SH, et al. Evolution of tricuspid valve regurgitation after implantation of a leadless pacemaker: A single center experience, systematic review, and meta-analysis. J Cardiovasc Electrophysiol. 2022;33(7):1617-27.

21. Hai JJ, Mao Y, Zhen Z, Fang J, Wong CK, Siu CW, et al. Close Proximity of Leadless Pacemaker to Tricuspid Annulus Predicts Worse Tricuspid Regurgitation Following Septal Implantation. Circ Arrhythm Electrophysiol. 2021;14(5):e009530.

22. Hasumi E, Fujiu K, Kawata T, Komuro I. The influence of His bundle pacing on tricuspid valve functioning using three-dimensional echocardiography. HeartRhythm Case Rep. 2018;4(9):437-8.

23. Hemayat S, Shafiee A, Oraii S, Roshanali F, Alaedini F, Aldoboni AS. Development of mitral and tricuspid regurgitation in right ventricular apex versus right ventricular outflow tract pacing. J Interv Card Electrophysiol. 2014;40(1):81-6.

24. Höke U, Auger D, Thijssen J, Wolterbeek R, van der Velde ET, Holman ER, et al. Significant lead-induced tricuspid regurgitation is associated with poor prognosis at long-term follow-up. Heart. 2014;100(12):960-8.

25. Jin QQ, Zheng C, Wang YJ, Lin JX, Wu DZ, Lin JF, et al. Feasibility of Left Bundle Branch Area Pacing Combined with Atrioventricular Node Ablation in Atrial Fibrillation Patients with Heart Failure. J Cardiovasc Dev Dis. 2022;9(10).

26. Kanawati J, Ng ACC, Khan H, Yu C, Hyun K, Abed H, et al. Long-Term Follow-Up of Mortality and Heart Failure Hospitalisation in Patients With Intracardiac Device-Related Tricuspid Regurgitation. Heart Lung Circ. 2021;30(5):692-7.

27. Kim JB, Spevack DM, Tunick PA, Bullinga JR, Kronzon I, Chinitz LA, et al. The effect of transvenous pacemaker and implantable cardioverter defibrillator lead placement on tricuspid valve function: an observational study. J Am Soc Echocardiogr. 2008;21(3):284-7.

28. Klutstein M, Balkin J, Butnaru A, Ilan M, Lahad A, Rosenmann D. Tricuspid incompetence following permanent pacemaker implantation. Pacing Clin Electrophysiol. 2009;32 Suppl 1:S135-7.

29. Kucukarslan N, Kirilmaz A, Ulusoy E, Yokusoglu M, Gramatnikovski N, Ozal E, et al. Tricuspid Insufficiency Does Not Increase Early After Permanent Implantation of Pacemaker Leads. Journal of Cardiac Surgery. 2006;21(4):391-4.

30. Lee RC, Friedman SE, Kono AT, Greenberg ML, Palac RT. Tricuspid Regurgitation Following Implantation of Endocardial Leads: Incidence and Predictors. Pacing Clin Electrophysiol. 2015;38(11):1267-74.

31. Lee WC, Fang HY, Chen HC, Chen YL, Tsai TH, Pan KL, et al. Progressive tricuspid regurgitation and elevated pressure gradient after transvenous permanent pacemaker implantation. Clin Cardiol. 2021;44(8):1098-105.

32. Leibowitz DW, Rosenheck S, Pollak A, Geist M, Gilon D. Transvenous pacemaker leads do not worsen tricuspid regurgitation: a prospective echocardiographic study. Cardiology. 2000;93(1-2):74-7.

33. Livesay J, Fogelson B, Tahir H, Baljepally R. Comparison of Tricuspid Regurgitation Severity Between Cardiac Resynchronization Therapy Versus Right Ventricular Pacing in Patients With Chronic Obstructive Pulmonary Disease. Cardiol Res. 2022;13(3):128-34.

34. Mao Y, Liu Y, Meng X, Ma Y, Li L, Zhai M, et al. Treatment of severe tricuspid regurgitation induced by permanent pacemaker lead: Transcatheter tricuspid valve replacement with the guidance of 3-dimensional printing. Front Cardiovasc Med. 2023;10:1030997.

35. Marincheva G, Levi T, Perelshtein Brezinov O, Valdman A, Rahkovich M, Kogan Y, et al. Echocardiography-guided Cardiac Implantable Electronic Device Implantation to Reduce Device Related Tricuspid Regurgitation: A Prospective Controlled Study. Isr Med Assoc J. 2022;24(1):25-32.

36. Markman TM, Kuo L, Mustin E, Amankwah NA, Supple GE, Dixit S, et al. Time Course and Predictors of Worsening Tricuspid Regurgitation Following Right Ventricular Lead Implantation. Circ Arrhythm Electrophysiol. 2020;13(11):e009177.

37. Nakajima H, Seo Y, Ishizu T, Iida N, Sato K, Yamamoto M, et al. Features of Lead-Induced Tricuspid Regurgitation in Patients With Heart Failure Events After Cardiac Implantation of Electronic Devices　- A Three-Dimensional Echocardiographic Study. Circ J. 2020;84(12):2302-11.

38. Papageorgiou N, Falconer D, Wyeth N, Lloyd G, Pellerin D, Speechly-Dick E, et al. Effect of tricuspid regurgitation and right ventricular dysfunction on long-term mortality in patients undergoing cardiac devices implantation: >10-year follow-up study. Int J Cardiol. 2020;319:52-6.

39. Riesenhuber M, Spannbauer A, Gwechenberger M, Pezawas T, Schukro C, Stix G, et al. Pacemaker lead-associated tricuspid regurgitation in patients with or without pre-existing right ventricular dilatation. Clin Res Cardiol. 2021;110(6):884-94.

40. Rothschild DP, Goldstein JA, Kerner N, Abbas AE, Patel M, Wong WS. Pacemaker-induced tricuspid regurgitation is uncommon immediately post-implantation. J Interv Card Electrophysiol. 2017;49(3):281-7.

41. Sadreddini M, Haroun MJ, Buikema L, Morillo C, Ribas S, Divakaramenon S, et al. Tricuspid valve regurgitation following temporary or permanent endocardial lead insertion, and the impact of cardiac resynchronization therapy. Open Cardiovasc Med J. 2014;8:113-20.

42. Saito M, Iannaccone A, Kaye G, Negishi K, Kosmala W, Marwick TH. Effect of Right Ventricular Pacing on Right Ventricular Mechanics and Tricuspid Regurgitation in Patients With High-Grade Atrioventricular Block and Sinus Rhythm (from the Protection of Left Ventricular Function During Right Ventricular Pacing Study). Am J Cardiol. 2015;116(12):1875-82.

43. Salaun E, Tovmassian L, Simonnet B, Giorgi R, Franceschi F, Koutbi-Franceschi L, et al. Right ventricular and tricuspid valve function in patients chronically implanted with leadless pacemakers. Europace. 2018;20(5):823-8.

44. Schleifer JW, Pislaru SV, Lin G, Powell BD, Espinosa R, Koestler C, et al. Effect of ventricular pacing lead position on tricuspid regurgitation: A randomized prospective trial. Heart Rhythm. 2018;15(7):1009-16.

45. Seo J, Kim DY, Cho I, Hong GR, Ha JW, Shim CY. Prevalence, predictors, and prognosis of tricuspid regurgitation following permanent pacemaker implantation. PLoS One. 2020;15(6):e0235230.

46. Seo Y, Nakajima H, Ishizu T, Iida N, Sato K, Yamamoto M, et al. Comparison of Outcomes in Patients With Heart Failure With Versus Without Lead-Induced Tricuspid Regurgitation After Cardiac Implantable Electronic Devices Implantations. Am J Cardiol. 2020;130:85-93.

47. Shan P, Su L, Zhou X, Wu S, Xu L, Xiao F, et al. Beneficial effects of upgrading to His bundle pacing in chronically paced patients with left ventricular ejection fraction <50. Heart Rhythm. 2018;15(3):405-12.

48. Stassen J, Galloo X, Hirasawa K, Marsan NA, van der Bijl P, Delgado V, et al. Tricuspid regurgitation after cardiac resynchronization therapy: evolution and prognostic significance. Europace. 2022;24(8):1291-9.

49. Vaidya VR, Dai M, Asirvatham SJ, Rea RF, Thome TM, Srivathsan K, et al. Real-world experience with leadless cardiac pacing. Pacing Clin Electrophysiol. 2019;42(3):366-73.

50. Van De Heyning CM, Elbarasi E, Masiero S, Brambatti M, Ghazal S, Al-Maashani S, et al. Prospective Study of Tricuspid Regurgitation Associated With Permanent Leads After Cardiac Rhythm Device Implantation. Can J Cardiol. 2019;35(4):389-95.

51. Vaturi M, Kusniec J, Shapira Y, Nevzorov R, Yedidya I, Weisenberg D, et al. Right ventricular pacing increases tricuspid regurgitation grade regardless of the mechanical interference to the valve by the electrode. Eur J Echocardiogr. 2010;11(6):550-3.

52. Wang N, Zhu T, Li Y, Cheng G, Chen Y, Fu Y, et al. His-Purkinje system pacing reduced tricuspid regurgitation in patients with persistent atrial fibrillation after left-sided valve surgery. Front Cardiovasc Med. 2023;10:1049482.

53. Webster G, Margossian R, Alexander ME, Cecchin F, Triedman JK, Walsh EP, et al. Impact of transvenous ventricular pacing leads on tricuspid regurgitation in pediatric and congenital heart disease patients. J Interv Card Electrophysiol. 2008;21(1):65-8.

54. Wiechecka K, Wiechecki B, Kapłon-Cieślicka A, Tymińska A, Budnik M, Hołowaty D, et al. Echocardiographic assessment of tricuspid regurgitation and pericardial effusion after cardiac device implantation. Cardiol J. 2020;27(6):797-806.

55. Wu S, Su L, Vijayaraman P, Zheng R, Cai M, Xu L, et al. Left Bundle Branch Pacing for Cardiac Resynchronization Therapy: Nonrandomized On-Treatment Comparison With His Bundle Pacing and Biventricular Pacing. Can J Cardiol. 2021;37(2):319-28.

56. Yu YJ, Chen Y, Lau CP, Liu YX, Wu MZ, Chen YY, et al. Nonapical Right Ventricular Pacing Is Associated with Less Tricuspid Valve Interference and Long-Term Progress of Tricuspid Regurgitation. J Am Soc Echocardiogr. 2020;33(11):1375-83.

57. Zhang W, Chen L, Zhou X, Huang J, Zhu S, Shen E, et al. Resynchronization effects and clinical outcomes during left bundle branch area pacing with and without conduction system capture. Clin Cardiol. 2023;46(3):287-95.

58. Achilli A, Peraldo C, Sassara M, Orazi S, Bianchi S, Laurenzi F, et al. Prediction of response to cardiac resynchronization therapy: The selection of candidates for CRT (SCART) study. PACE - Pacing and Clinical Electrophysiology. 2006;29(SUPPL. 2):S11-S9.

59. Achilli A, Sassara M, Pontillo D, Turreni F, Rossi P, De Luca R, et al. Effectiveness of cardiac resynchronisation therapy in patients with echocardiographic evidence of mechanical dyssynchrony. Journal of Cardiovascular Medicine. 2008;9(2):131-6.

60. Adelstein E, Saba S. Right atrial pacing and the risk of postimplant atrial fibrillation in cardiac resynchronization therapy recipients. American Heart Journal. 2008;155(1):94-9.

61. António N, Lourenço C, Teixeira R, Saraiva F, Coelho L, Ventura M, et al. Cardiac resynchronization therapy is effective even in elderly patients with comorbidities. Journal of Interventional Cardiac Electrophysiology. 2010;27(1):61-8.

62. Atta S, Bashandy M, Zaky S. Baseline QRS width and mitral regurgitation behavior after cardiac resynchronization therapy among patients with dilated cardiomyopathy. Egyptian Heart Journal. 2014;66(4):335-42.

63. Bakker PF, Meijburg HW, de Vries JW, Mower MM, Thomas AC, Hull ML, et al. Biventricular pacing in end-stage heart failure improves functional capacity and left ventricular function. J Interv Card Electrophysiol. 2000;4(2):395-404.

64. Beaudoin J, Singh JP, Szymonifka J, Zhou Q, Levine RA, Januzzi JL, et al. Novel Heart Failure Biomarkers Predict Improvement of Mitral Regurgitation in Patients Receiving Cardiac Resynchronization Therapy-The BIOCRT Study. Can J Cardiol. 2016;32(12):1478-84.

65. Binda C, Menet A, Appert L, Ennezat PV, Delelis F, Castel AL, et al. Time course of secondary mitral regurgitation in patients with heart failure receiving cardiac resynchronization therapy: Impact on long-term outcome beyond left ventricular reverse remodelling. Arch Cardiovasc Dis. 2018;111(5):320-31.

66. Bordachar P, Lafitte S, Reuter S, Garrigue S, Sanders P, Roudaut R, et al. Biventricular pacing and left ventricular pacing in heart failure: similar hemodynamic improvement despite marked electromechanical differences. J Cardiovasc Electrophysiol. 2004;15(12):1342-7.

67. Boriani G, Gasparini M, Landolina M, Lunati M, Biffi M, Santini M, et al. Impact of mitral regurgitation on the outcome of patients treated with CRT-D: Data from the InSync ICD Italian registry. PACE - Pacing and Clinical Electrophysiology. 2012;35(2):146-54.

68. Brandt RR, Reiner C, Arnold R, Sperzel J, Pitschner HF, Hamm CW. Contractile response and mitral regurgitation after temporary interruption of long-term cardiac resynchronization therapy. European Heart Journal. 2006;27(2):187-92.

69. Breithardt OA, Sinha AM, Schwammenthal E, Bidaoui N, Markus KU, Franke A, et al. Acute effects of cardiac resynchronization therapy on functional mitral regurgitation in advanced systolic heart failure. Journal of the American College of Cardiology. 2003;41(5):765-70.

70. Brzezińska B, Łoboz-Grudzień K, Wita K, Mizia-Stec K, Gasior Z, Kasprzak JD, et al. Predictors of functional mitral regurgitation improvement during a short-term follow-up after cardiac resynchronisation therapy. Kardiologia Polska. 2016;74(7):665-73.

71. Cabrera-Bueno F, Molina-Mora MJ, Alzueta J, Pena-Hernandez J, Jimenez-Navarro M, Fernandez-Pastor J, et al. Persistence of secondary mitral regurgitation and response to cardiac resynchronization therapy. European Journal of Echocardiography. 2010;11(2):131-7.

72. Chatterjee NA, Gold MR, Waggoner AD, Picard MH, Stein KM, Yu Y, et al. Longer left ventricular electric delay reduces mitral regurgitation after cardiac resynchronization therapy. Circulation: Arrhythmia and Electrophysiology. 2016;9(11).

73. Cipriani M, Lunati M, Landolina M, Proclemer A, Boriani G, Ricci RP, et al. Prognostic implications of mitral regurgitation in patients after cardiac resynchronization therapy. European Journal of Heart Failure. 2016;18(8):1060-8.

74. Cleland J, Freemantle N, Ghio S, Fruhwald F, Shankar A, Marijanowski M, et al. Predicting the long-term effects of cardiac resynchronization therapy on mortality from baseline variables and the early response a report from the CARE-HF (Cardiac Resynchronization in Heart Failure) Trial. Journal of the American College of Cardiology. 2008;52(6):438‐45.

75. Delnoy PP, Ottervanger JP, Luttikhuis HO, Elvan A, Misier AR, Beukema WP, et al. Comparison of usefulness of cardiac resynchronization therapy in patients with atrial fibrillation and heart failure versus patients with sinus rhythm and heart failure. Am J Cardiol. 2007;99(9):1252-7.

76. Di Biase L, Auricchio A, Mohanty P, Bai R, Kautzner J, Pieragnoli P, et al. Impact of cardiac resynchronization therapy on the severity of mitral regurgitation. Europace. 2011;13(6):829-38.

77. Donnellan E, Wazni OM, Saliba WI, Baranowski B, Hanna M, Martyn M, et al. Cardiac devices in patients with transthyretin amyloidosis: Impact on functional class, left ventricular function, mitral regurgitation, and mortality. J Cardiovasc Electrophysiol. 2019;30(11):2427-32.

78. Ennezat PV, Gal B, Kouakam C, Marquie C, LeTourneau T, Klug D, et al. Cardiac resynchronisation therapy reduces functional mitral regurgitation during dynamic exercise in patients with chronic heart failure: An acute echocardiographic study. Heart. 2006;92(8):1091-5.

79. Fukuda S, Grimm R, Song JM, Kihara T, Daimon M, Agler DA, et al. Electrical conduction disturbance effects on dynamic changes of functional mitral regurgitation. Journal of the American College of Cardiology. 2005;46(12):2270-6.

80. Gras D, Leclercq C, Tang AS, Bucknall C, Luttikhuis HO, Kirstein-Pedersen A. Cardiac resynchronization therapy in advanced heart failure the multicenter InSync clinical study. Eur J Heart Fail. 2002;4(3):311-20.

81. Iori M, Bottoni N, Quartieri F, Manari A. E/A ratio before cardiac resynchronization therapy predicts left ventricle reverse remodeling. Minerva Cardioangiologica. 2014;62(4):305-9.

82. Jin H, Yang S, Hua W, Gu M, Niu H, Ding L, et al. Significant mitral regurgitation as a predictor of long-term prognosis in patients receiving cardiac resynchronisation therapy. Kardiologia Polska. 2018;76(6):987-92.

83. Kanzaki H, Bazaz R, Schwartzman D, Dohi K, Sade LE, Gorcsan J, 3rd. A mechanism for immediate reduction in mitral regurgitation after cardiac resynchronization therapy: insights from mechanical activation strain mapping. J Am Coll Cardiol. 2004;44(8):1619-25.

84. Karaca O, Cakal B, Omaygenc MO, Gunes HM, Kizilirmak F, Cakal SD, et al. Effect of cardiac resynchronization therapy on mitral valve geometry: a novel aspect as "reversed mitral remodeling". International Journal of Cardiovascular Imaging. 2018;34(7):1029-40.

85. Karvounis HI, Dalamaga EG, Papadopoulos CE, Karamitsos TD, Vassilikos V, Paraskevaidis S, et al. Improved papillary muscle function attenuates functional mitral regurgitation in patients with dilated cardiomyopathy after cardiac resynchronization therapy. Journal of the American Society of Echocardiography. 2006;19(9):1150‐7.

86. Kelarijani RB, Dadjoo Y, Chalian H, Naserbakht M, Kabir K, Saleh DK. Over-time mitral regurgitation changes following cardiac resynchronization therapy. Advances in medical sciences. 2008;53:94-8.

87. Killu AM, Wu JH, Friedman PA, Shen WK, Webster TL, Brooke KL, et al. Outcomes of cardiac resynchronization therapy in the elderly. PACE - Pacing and Clinical Electrophysiology. 2013;36(6):664-72.

88. Kranig W, Grove RG, Wolff EW, Kowalski MK, Thale JT. Multiple BIV stimulation combinations by using two RV leads improve potential for response to CRT: results of the TRIV HF ICD study. Europace. 2015;17:iii79.

89. Kuppahally SS, Fowler MB, Vagelos R, Wang P, Al-Ahmad A, Paloma A, et al. Worsening of left ventricular end-systolic volume and mitral regurgitation without increase in left ventricular dyssynchrony on acute interruption of cardiac resynchronization therapy. Echocardiography (mount kisco, NY). 2009;26(7):759‐65.

90. Lancellotti P, Melon P, Sakalihasan N, Waleffe A, Dubois C, Bertholet M, et al. Effect of cardiac resynchronization therapy on functional mitral regurgitation in heart failure. Am J Cardiol. 2004;94(11):1462-5.

91. Lau CP, Yu CM, Chau E, Fan K, Tse HF, Lee K, et al. Reversal of left ventricular remodeling by synchronous biventricular pacing in heart failure. PACE - Pacing and Clinical Electrophysiology. 2000;23(11 II):1722-5.

92. Liang YJ, Zhang Q, Fung JW, Chan JY, Yip GW, Lam YY, et al. Different determinants of improvement of early and late systolic mitral regurgitation contributed after cardiac resynchronization therapy. J Am Soc Echocardiogr. 2010;23(11):1160-7.

93. Linde C, Leclercq C, Rex S, Garrigue S, Lavergne T, Cazeau S, et al. Long-term benefits of biventricular pacing in congestive heart failure: Results from the MUltisite STimulation In Cardiomyopathy (MUSTIC) study. Journal of the American College of Cardiology. 2002;40(1):111-8.

94. Madaric J, Vanderheyden M, Van Laethem C, Verhamme K, Feys A, Goethals M, et al. Early and late effects of cardiac resynchronization therapy on exercise-induced mitral regurgitation: Relationship with left ventricular dyssynchrony, remodelling and cardiopulmonary performance. European Heart Journal. 2007;28(17):2134-41.

95. Mangiavacchi M, Gasparini M, Faletra F, Klersy C, Morenghi E, Galimberti P, et al. Clinical predictors of marked improvement in left ventricular performance after cardiac resynchronization therapy in patients with chronic heart failure. American Heart Journal. 2006;151(2):477.e1-.e6.

96. Marechaux S, Pincon C, Gal B, Kouakam C, Marquie C, Lacroix D, et al. Functional mitral regurgitation at rest determines the acute hemodynamic response to cardiac resynchronization therapy during exercise: an acute exercise echocardiographic study. J Am Soc Echocardiogr. 2009;22(5):464-71.

97. Martens P, Verbrugge FH, Bertrand PB, Verhaert D, Vandervoort P, Dupont M, et al. Effect of Cardiac Resynchronization Therapy on Exercise-Induced Pulmonary Hypertension and Right Ventricular-Arterial Coupling. Circulation Cardiovascular imaging. 2018;11(9):e007813.

98. Matsumoto K, Tanaka H, Okajima K, Hayashi T, Kajiya T, Kawai H, et al. Relation between left ventricular morphology and reduction in functional mitral regurgitation by cardiac resynchronization therapy in patients with idiopathic dilated cardiomyopathy. American Journal of Cardiology. 2011;108(9):1327-34.

99. Menardi E, Vado A, Rossetti G, Racca E, Conte E, Deorsola A, et al. Cardiac Resynchronization Therapy Modifies the Neurohormonal Profile, Hemodynamic and Functional Capacity in Heart Failure Patients. Archives of Medical Research. 2008;39(7):702-8.

100. Mihos CG, Yucel E, Capoulade R, Orencole MP, Upadhyay GA, Santana O, et al. Impact of cardiac resynchronization therapy on mitral valve apparatus geometry and clinical outcomes in patients with secondary mitral regurgitation. Echocardiography. 2017;34(11):1561-7.

101. Molhoek SG, Bax JJ, Van Erven L, Bootsma M, Boersma E, Steendijk P, et al. Comparison of benefits from cardiac resynchronization therapy in patients with ischemic cardiomyopathy versus idiopathic dilated cardiomyopathy. American Journal of Cardiology. 2004;93(7):860-3.

102. Naqvi TZ, Rafique AM, Swerdlow C, Verma S, Siegel RJ, Tolstrup K, et al. Predictors of reduction in mitral regurgitation in patients undergoing cardiac resynchronisation treatment. Heart. 2008;94(12):1580-8.

103. Onishi T, Onishi T, Marek JJ, Ahmed M, Haberman SC, Oyenuga O, et al. Mechanistic features associated with improvement in mitral regurgitation after cardiac resynchronization therapy and their relation to long-term patient outcome. Circ Heart Fail. 2013;6(4):685-93.

104. Pfau G, Schilling T, Kozian A, Lux A, Götte A, Huth C, et al. Outcome After Implantation of Cardiac Resynchronization/Defibrillation Systems in Patients With Congestive Heart Failure and Left Bundle-Branch Block. Journal of Cardiothoracic and Vascular Anesthesia. 2010;24(1):30-6.

105. Porciani MC, Macioce R, Demarchi G, Chiostri M, Musilli N, Cappelli F, et al. Effects of cardiac resynchronization therapy on the mechanisms underlying functional mitral regurgitation in congestive heart failure. European Journal of Echocardiography. 2006;7(1):31-9.

106. Praus R, Haman L, Tauchman M, Pudil R, Blaha V, Parizek P. Echocardiographic changes after cardiac resynchronisation therapy. Kardiologia Polska. 2012;70(12):1250-7.

107. Rao RK, Kumar UN, Schafer J, Viloria E, De Lurgio D, Foster E. Reduced ventricular volumes and improved systolic function with cardiac resynchronization therapy: a randomized trial comparing simultaneous biventricular pacing, sequential biventricular pacing, and left ventricular pacing. Circulation. 2007;115(16):2136-44.

108. Reuter S, Garrigue S, Bordachar P, Hocini M, Jais P, Haissaguerre M, et al. Intermediate-term results of biventricular pacing in heart failure: Correlation between clinical and hemodynamic data. PACE - Pacing and Clinical Electrophysiology. 2000;23(11 II):1713-7.

109. Rocha EA, Pereira FT, Abreu JS, Lima JW, Monteiro Mde P, Rocha Neto AC, et al. Echocardiographic Predictors of Worse Outcome After Cardiac Resynchronization Therapy. Arq Bras Cardiol. 2015;105(6):552-9.

110. Sassone B, De Simone N, Parlangeli G, Tortorici R, Biancoli S, Di Pasquale G. Pacemaker-induced mitral regurgitation: prominent role of abnormal ventricular activation sequence versus altered atrioventricular synchrony. Ital Heart J. 2001;2(6):441-8.

111. Sénéchal M, Lancellotti P, Magne J, Garceau P, Champagne J, Philippon F, et al. Impact of Mitral Regurgitation and Myocardial Viability on Left Ventricular Reverse Remodeling After Cardiac Resynchronization Therapy in Patients With Ischemic Cardiomyopathy. American Journal of Cardiology. 2010;106(1):31-7.

112. Sitges M, Vidal B, Delgado V, Mont L, Garcia-Alvarez A, Tolosana JM, et al. Long-Term Effect of Cardiac Resynchronization Therapy on Functional Mitral Valve Regurgitation. American Journal of Cardiology. 2009;104(3):383-8.

113. Solis J, McCarty D, Levine RA, Handschumacher MD, Fernandez-Friera L, Chen-Tournoux A, et al. Mechanism of decrease in mitral regurgitation after cardiac resynchronization therapy: optimization of the force-balance relationship. Circ Cardiovasc Imaging. 2009;2(6):444-50.

114. Solomon SD, Foster E, Bourgoun M, Shah A, Viloria E, Brown MW, et al. Effect of cardiac resynchronization therapy on reverse remodeling and relation to outcome: multicenter automatic defibrillator implantation trial: cardiac resynchronization therapy. Circulation. 2010;122(10):985-92.

115. Stellbrink C, Breithardt OA, Franke A, Sack S, Bakker P, Auricchio A, et al. Impact of cardiac resynchronization therapy using hemodynamically optimized pacing on left ventricular remodeling in patients with congestive heart failure and ventricular conduction disturbances. Journal of the American College of Cardiology. 2001;38(7):1957‐65.

116. St John Sutton MG, Plappert T, Hilpisch KE, Abraham WT, Hayes DL, Chinchoy E. Sustained reverse left ventricular structural remodeling with cardiac resynchronization at one year is a function of etiology: Quantitative Doppler echocardiographic evidence from the Multicenter InSync Randomized Clinical Evaluation (MIRACLE). Circulation. 2006;113(2):266-72.

117. St John Sutton M, Ghio S, Plappert T, Tavazzi L, Scelsi L, Daubert C, et al. Cardiac resynchronization induces major structural and functional reverse remodeling in patients with New York Heart Association class I/II heart failure. Circulation. 2009;120(19):1858-65.

118. Stockburger M, Nitardy A, Fateh-Moghadam S, Krebs A, Celebi O, Karhausen T, et al. Electrical remodeling and cardiac dimensions in patients treated by cardiac resynchronization and heart failure controls. PACE - Pacing and Clinical Electrophysiology. 2008;31(1):70-7.

119. Stolfo D, Tonet E, Barbati G, Gigli M, Pinamonti B, Zecchin M, et al. Acute Hemodynamic Response to Cardiac Resynchronization in Dilated Cardiomyopathy: Effect on Late Mitral Regurgitation. PACE - Pacing and Clinical Electrophysiology. 2015;38(11):1287-96.

120. Tournoux FB, Alabiad C, Fan D, Chen AA, Chaput M, Heist EK, et al. Echocardiographic measures of acute haemodynamic response after cardiac resynchronization therapy predict long-term clinical outcome. European Heart Journal. 2007;28(9):1143-8.

121. Upadhyay GA, Chatterjee NA, Kandala J, Friedman DJ, Park MY, Tabtabai SR, et al. Assessing mitral regurgitation in the prediction of clinical outcome after cardiac resynchronization therapy. Heart Rhythm. 2015;12(6):1201-8.

122. Upadhyay GA, Henry M, Genovese D, Desai P, Lattell J, Wey H, et al. Impact of physiological pacing on functional mitral regurgitation in systolic dysfunction: Initial echocardiographic remodeling findings after His bundle pacing. Heart Rhythm O2. 2021;2(5):446-54.

123. van Bommel RJ, Marsan NA, Delgado V, Borleffs CJ, van Rijnsoever EP, Schalij MJ, et al. Cardiac resynchronization therapy as a therapeutic option in patients with moderate-severe functional mitral regurgitation and high operative risk. Circulation. 2011;124(8):912-9.

124. van der Bijl P, Khidir M, Ajmone Marsan N, Delgado V, Leon MB, Stone GW, et al. Effect of Functional Mitral Regurgitation on Outcome in Patients Receiving Cardiac Resynchronization Therapy for Heart Failure. American Journal of Cardiology. 2019;123(1):75-83.

125. Verhaert D, Popović ZB, De S, Puntawangkoon C, Wolski K, Wilkoff BL, et al. Impact of mitral regurgitation on reverse remodeling and outcome in patients undergoing cardiac resynchronization therapy. Circulation: Cardiovascular Imaging. 2012;5(1):21-6.

126. Vinereanu D, Turner MS, Bleasdale RA, Mumford CE, Cinteza M, Frenneaux MP, et al. Mechanisms of Reduction of Mitral Regurgitation by Cardiac Resynchronization Therapy. Journal of the American Society of Echocardiography. 2007;20(1):54-62.

127. Witte KK, Sasson Z, Persaud JA, Jolliffe R, Wald RW, Parker JD. Biventricular pacing: Impact on exercise-induced increases in mitral insufficiency in patients with chronic heart failure. Canadian Journal of Cardiology. 2008;24(5):379-84.

128. Ypenburg C, Lancellotti P, Tops LF, Boersma E, Bleeker GB, Holman ER, et al. Mechanism of improvement in mitral regurgitation after cardiac resynchronization therapy. European Heart Journal. 2008;29(6):757-65.

129. Yu CM, Chan YS, Zhang Q, Yip GWK, Chan CK, Kum LCC, et al. Benefits of Cardiac Resynchronization Therapy for Heart Failure Patients With Narrow QRS Complexes and Coexisting Systolic Asynchrony by Echocardiography. Journal of the American College of Cardiology. 2006;48(11):2251-7.

130. Zanon F, Aggio S, Baracca E, Bilato C, Corbucci G, Rigatelli G, et al. Reduced mitral regurgitation in heart failure patients submitted to cardiac resynchronization therapy: a short-term prospective study. Italian heart journal : official journal of the Italian Federation of Cardiology. 2004;5(11):826-30.
